# New Hydroquinone Monoterpenoid and Cembranoid-Related Metabolites from the Soft Coral *Sarcophyton tenuispiculatum*

**DOI:** 10.3390/md19010008

**Published:** 2020-12-27

**Authors:** Tzu-Yin Huang, Chiung-Yao Huang, Shu-Rong Chen, Jing-Ru Weng, Tzu-Hsuan Tu, Yuan-Bin Cheng, Shih-Hsiung Wu, Jyh-Horng Sheu

**Affiliations:** 1 Doctoral Degree Program in Marine Biotechnology, National Sun Yat-Sen University, Kaohsiung 804, Taiwan; HuangTY@g-mail.nsysu.edu.tw (T.-Y.H.); jrweng@mail.nsysu.edu.tw (J.-R.W.); 2 Department of Marine Biotechnology and Resources, National Sun Yat-sen University, Kaohsiung 804, Taiwan; huangcy@mail.nsysu.edu.tw (C.-Y.H.); jmb@kmu.edu.tw (Y.-B.C.); 3 Graduate Institute of Natural Products, College of Pharmacy, Kaohsiung Medical University, Kaohsiung 807, Taiwan; u106831002@kmu.edu.tw; 4 Department of Oceanography, National Sun Yat-sen University, Kaohsiung 804, Taiwan; thtu@mail.nsysu.edu.tw; 5 Institute of Biological Chemistry, Academia Sinica, Taipei 11529, Taiwan; shwu@gate.sinica.edu.tw; 6 Department of Medical Research, China Medical University Hospital, China Medical University, Taichung 404, Taiwan

**Keywords:** *Sarcophyton tenuispiculatum*, hydroquinone, cembranoid, anti-inflammatory, cytotoxicity, LPS-stimulated J774A.1 macrophage cells, PPAR-γ transcription factor

## Abstract

Chemical investigation of the marine soft coral *Sarcophyton tenuispiculatum* resulted in the isolation of a 1,4-dihydrobenzoquinone, sarcotenuhydroquinone (**1**), three new cembranoids, sarcotenusenes A‒C (**2**‒**4**), and ten previously reported metabolites **5**–**14**. The chemical structures of all isolated metabolites were determined by detailed spectroscopic analyses. In biological assays, anti-inflammatory, cytotoxic, and peroxisome proliferator-activated receptor γ (PPAR-γ) transcription factor assays of all compounds were performed. None of the isolated compounds were found to exhibit activity in the PPAR-γ transcription factor assay. The anti-inflammatory assays showed that (+)-7*α*,8*β*-dihydroxydeepoxysarcophine (**13**) inhibited the production of IL-1*β* to 56 ± 1% at a concentration of 30 *µ*M in lipopolysaccharide (LPS)-stimulated J774A.1 macrophage cells. In addition, **1** and **2** were found to exhibit cytotoxicity towards a panel of cancer cell lines.

## 1. Introduction

**Figure 1:**
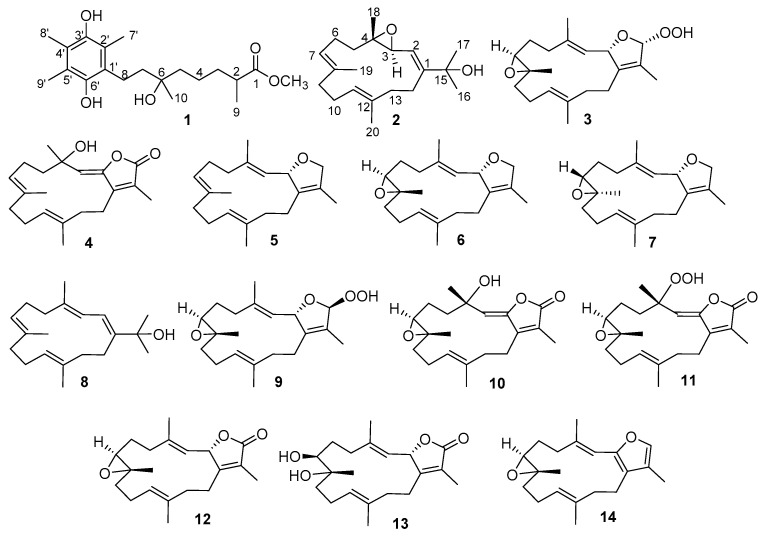
Compounds **1**–**14** isolated from *S. tenuispiculatum.*

Marine organisms, especially sessile marine invertebrates, such as soft corals, have been found to produce secondary metabolites as defensive substances to protect themselves from predators or stressful environments [1]. *Sarcophyton*, one of the widely studied genera of soft corals, is considered an abundant source of bioactive diterpenes, especially of cembranoids. For example, cembranoid-type metabolite sarcophine, isolated from the marine soft coral *Sarcophyton glaucum* in 1974 [2], was found to play an important role in the defense mechanism against environmental predators [3]. Subsequently, a series of analogs were discovered from different *Sarcophyton* species. Importantly, these compounds have been proven to display diverse biological activities, including cytotoxic [4,5,6,7,8,9,10], anti-inflammatory [11,12,13,14], and antiviral [15] activities. In our study of discovering bioactive natural products, we focused on the investigation of secondary metabolites obtained from the soft coral *Sarcophyton tenuispiculatum* and examined the biological activities of the isolates. Moreover, the lack of published studies focused on *S. tenuispiculatum* encouraged us to investigate this soft coral in detail. This study led to the discovery of 1,4-dihydrobenzoquinone, sarcotenuhydroquinone (**1**), three new cembranoids, sarcotenusenes A–C (**2**–**4**), and ten previously reported metabolites (**5**–**13**), including sarcophytonin A (**5**) [16], (2*S*, 7*S*, 8*S*)-sarcophytoxide (**6**) [16], (2*S*, 7*R*, 8*R*)-sarcophytoxide (**7**) [17], dehydronephthenol (**8**) [18], sarcophytonin F (**9**) [16], 3,4-dihydro-4*α*-hydroxy-Δ2-sarcophine (**10**) [19], a hydroperoxide obtained by autoxidation of dihydrofuranocembranoid (**11**) [20], (+)-sarcophine (**12**) [3], (+)-7*α*,8*β*-dihydroxydeepoxysarcophine (**13**) [6], and 2,16:7*S*, 8*S*-diepoxy-1,3,11,15-cembratetraene (**14**) [21]. All of these structures are shown in Figure 1.

Peroxisome proliferator-activated receptor-γ (PPAR-γ) is a nuclear protein involved in many biological processes, such as cellular differentiation, lipid metabolism, and insulin sensitization [22]. A study revealed that overexpression of PPAR-γ leads to the inhibition of various tumor cells and cell apoptosis via degradation of nuclear factor-*κ*B (NF-*κ*B) [23]. Therefore, to identify more PPAR-γ agonists for developing anti-cancer medicines, the biological activities, including cytotoxicity and PPAR-γ transcription factor assay activity, of all the isolated compounds were examined.

Furthermore, the anti-inflammatory activity of all metabolites was assessed by screening the production of pro-inflammatory cytokines in lipopolysaccharide (LPS)-stimulated murine macrophage J774A.1 cells. LPS is the major component of the outer membrane in gram-negative bacteria and stimulates macrophage cells to produce pro-inflammatory mediators, such as tumor necrosis factor *α* (TNF-*α*) and interleukin-1*β* (IL-1*β*) [24]. Recent research demonstrated that IL-1*β* plays an important role in therapeutic targets for auto-inflammatory diseases, such as familial Mediterranean fever, Schnitzler syndrome, and adult-onset Still′s disease [25]. Further, many common diseases, including rheumatoid arthritis [25,26,27], cardiovascular disease [25,28], heart failure [25], and type 2 diabetes [25,29], have been shown to be related to IL-1*β* production. Accordingly, the mediation of IL-1*β* production could assist in reducing the severity of these diseases. In the present study, none of the isolated metabolites exhibited activity in the PPAR-γ transcription factor assay, whereas in anti-inflammatory assays, (+)-7*α*,8*β*-dihydroxydeepoxysarcophine (13) exhibited inhibition of IL-1*β* production in LPS-stimulated J774A.1 macrophage cells. In addition, **1** and **2** exhibited cytotoxicity towards a panel of cancer cell lines.

## 2. Results and Discussion

Soft coral *S. tenuispiculatum* was sliced then exhaustedly extracted by acetone. The oily residue was continuously purified by column chromatography to obtain four new compounds **1**–**4** and ten previously reported metabolites **5**–**14**. The structures of all new compounds were determined by analyzing the IR, MS, 1D NMR, and 2D NMR spectra (Supplementary Materials Figures S1–S41). Furthermore, the ^13^C and ^1^H NMR spectroscopic data are listed in Table 1, Table 2 and Table 3.

Sarcotenuhydroquinone (**1**) was found to be a colorless oil. The molecular formula of **1**, C_20_H_32_O_5_, was deduced from the high-resolution electrospray ionization mass spectrometry (HRESIMS) spectrum (calculated: 375.2142; found: 375.2149, ${\left[M\;+\;\mathrm{Na}\right]}^{+}$). IR absorptions at 3434, 1726, and 1647 cm^−1^ indicated the presence of hydroxy, ester carbonyl, and olefinic groups, respectively. The ^13^C NMR (Table 1) spectrum showed signals of 20 carbons, which were identified from the distortionless enhancement by transfer (DEPT) spectrum as six methyl groups, five *sp^3^* methylenes, one *sp^3^* methine group, seven *sp^2^* quaternary carbons, and one *sp^3^* quaternary carbon. The ^1^H and^13^C NMR spectra of **1** showed signals of one methoxy group (*δ*_H_ 3.66, s; *δ*_C_ 51.5), three olefinic methyl groups (*δ*_H_ 2.16, s; 2.11, s; 2.10, s, and *δ*_C_ 12.2, 11.3, 11.8, respectively), one methyl singlet (*δ*_H_ 1.21, s; *δ*_C_ 23.7), one methyl doublet (*δ*_H_ 1.14, d, *J* = 7.2 Hz; *δ*_C_ 17.0), one sextet methine (*δ*_H_ 2.46, sext, *J* = 6.8 Hz; *δ*_C_ 39.4), and three tetrasubstituted double bonds (*δ*_C_ 145.3, 144.6, 122.6, 121.0, 118.5, and 117.2).

**Figure 2:**
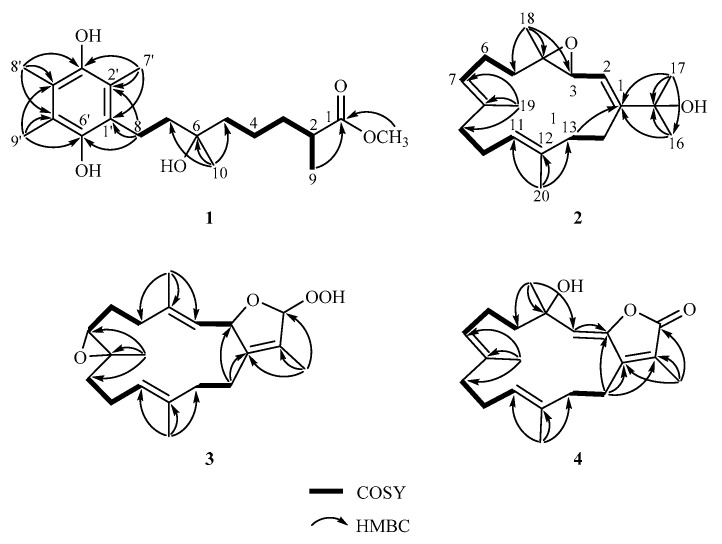
COSY and selective HMBC of **1**–**4**.

The planar structure of **1** was established by a detailed analysis of the 2D NMR spectra. The COSY data revealed two separate spin systems, from H_3_-9 via H-2, H_2_-3, H_2_-4 to H_2_-5 and H_2_-7 to H_2_-8. From the HMBC signals from H_3_-9 to C-1, C-2, and C-3; H_3_-10 to C-5, C-6, and C-7, a linear monoterpenoid functional group was deduced. In addition, the HMBC from both H-2 (*δ*_H_ 2.46, sext, *J* = 6.8 Hz) and the methoxy group (*δ*_H_ 3.66, s) to the ester carbonyl group (*δ*_C_ 177.3) indicated the presence of a methoxycarbonyl group at C-1. Furthermore, the structure of a hydroquinone moiety was established by HMBC correlations from H_3_-7′ to C-1′, C-2′, C-3′; H_3_-8′ to C-3′, C-4′, C-5′, and H_3_-9′ to C-4′, C-5′, C-6′. A hydroquinone moiety fused to the monoterpene structure was also determined from HMBC from H_2_-8 to C-1′, C-2′, C-6′ and from H_2_-7 to C-1′. The above evidence was used to establish the planar structure of **1** as a hydroquinone monoterpenoid, as shown in Figure 2, which was closely related to that of a previous metabolite, flexibiliquinone, isolated from the marine soft coral *Sinularia flexibilis* [30]. This was the first discovery of hydroquinone of 3,5,6-trimethyl-1,4-dihydroxybenzene substituted with a rare C_10_ side chain group of flexibiliquinone and this compound which was named sarcotenuhydroquinone (**1**).

Sarcotenusene A (**2**), with the molecular formula C_20_H_32_O_2_ determined by the HRESIMS spectrum (calculated: 305.2475; found: 305.2474, ${\left[M\;+\;H\right]}^{+}$), was isolated as a colorless oil. Its IR spectrum showed broad absorption at 3399 cm^−1^, suggesting the presence of a hydroxy group. The ^13^C NMR and DEPT (Table 2) spectra displayed 20 carbon signals, including five methyl groups, six methylenes, four methines, and five quaternary carbons. Accordingly, the ^1^H (Table 3) and ^13^C NMR spectra revealed two olefinic methyl groups (*δ*_H_ 1.64, s; *δ*_C_ 16.0 and *δ*_H_ 1.57, s; *δ*_C_ 15.9, respectively), two methyl groups linked to oxygen-bearing quaternary carbons (*δ*_H_ 1.37, s; *δ*_C_ 30.0 and *δ*_H_ 1.37, s; *δ*_C_ 29.9, respectively), one oxygen-bearing quaternary carbon (*δ*_C_ 73.7), one epoxy group (*δ*_H_ 3.42, d, *J* = 7.6 Hz; *δ*_C_ 58.5, CH, and 61.9, C), and three trisubstituted double bonds (*δ*_H_ 5.37, d, *J* = 7.6 Hz; *δ*_C_ 119.8, CH, 153.8, C; *δ*_H_ 5.07, t, *J* = 6.8 Hz; *δ*_C_ 125.1, CH, 135.1, C, and *δ*_H_ 5.00, t, *J* = 6.4 Hz; *δ*_C_ 125.4, CH, 134.5, C, respectively). The molecular structure of **2** was further determined by 2D NMR spectra (COSY and HMBC). The COSY spectrum showed four partial structures from H-2 to H-3, from H_2_-5 to H-7, from H_2_-9 to H-11, and from H_2_-13 to H_2_-14, respectively. These partial structures were connected by HMBC from H_3_-16 to C-1, C-15, and C-17; H_3_-18 to C-3, C-4, and C-5; H_3_-19 to C-7, C-8, and C-9; H_3_-20 to C-11, C-12, and C-13; and H_2_-13 to C-1 and C-14. The HMBC from H_3_-18 to C-3 (*δ*_C_ 58.5) and C-4 (*δ*_C_ 61.9) also revealed the presence of an epoxy group at C-3 and C-4. Accordingly, the planar structure of **2** was established as a cembranoid skeleton derived from dehydronephthenol (**8**) [18].

**Figure 3:**
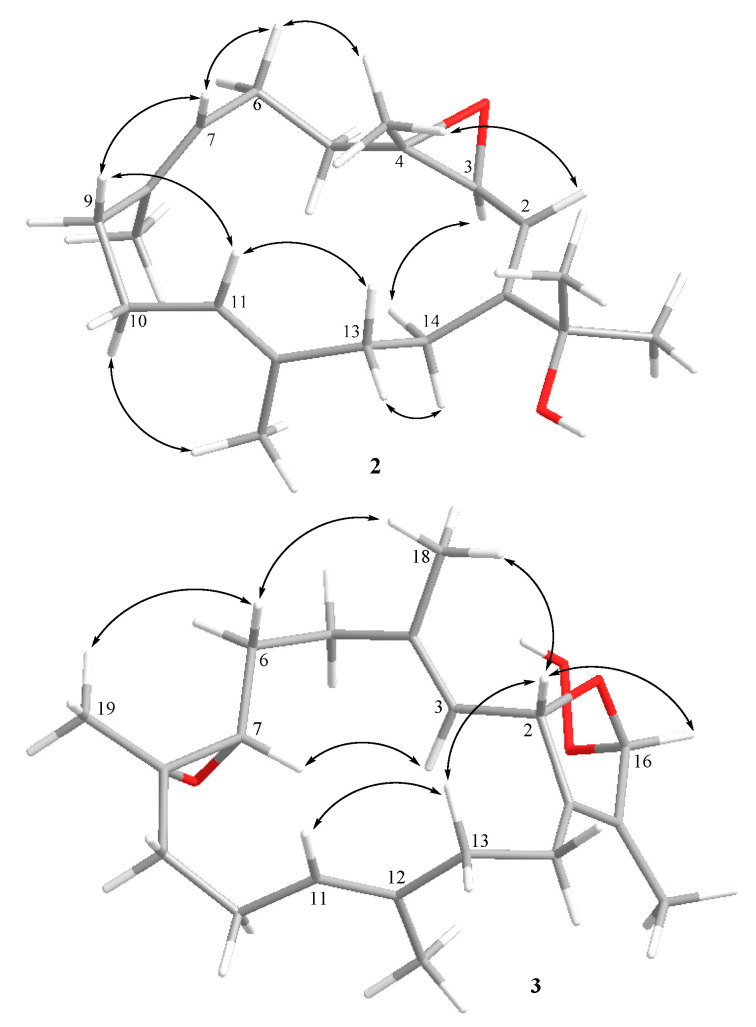
Selective NOESY correlations of **2** and **3.**

The relative configuration of **2** (Figure 3) was determined by detailed analyses of correlations recorded by nuclear Overhauser effect (NOE) spectroscopy (NOESY). Assuming the *β*-orientation of H_3_-18 (*δ*_H_ 1.28, s), NOE correlations of H-2 (*δ*_H_ 5.37, d, *J* = 7.6 Hz) and H_3_-18 rather than H-3 (*δ*_H_ 3.42, d, *J* = 7.6 Hz) were observed, suggesting the *α*-orientation of H-3. Furthermore, the upfield chemical shifts of C-19 (*δ*_C_ 15.9) and C-20 (*δ*_C_ 16.0) indicated the *E* geometry for both of the trisubstituted C-7/C-8 and C-11/C-12 double bonds. Consequently, the relative configuration of **2** was determined as 2R* and 3S*.

Sarcotenusene B (**3**) appeared to be a pale amorphous oil. The HRESIMS spectrum showed a sodiated molecular ion peak at m/z 357.2032 ${\left[M\;+\;\mathrm{Na}\right]}^{+}$ (calculated for C_20_H_30_O_4_Na: 357.2036), suggesting the molecular formula of C_20_H_30_O_4_. In the IR spectrum, absorptions at 3452 and 1670 cm^−1^ arose from hydroxy and olefinic functional groups. The ^13^C NMR and DEPT spectra showed signals of 20 carbons: four methyls, six *sp^3^* methylenes, three *sp^3^* methines, two *sp^2^* methines, an *sp^3^* quaternary carbon, and four *sp^2^* quaternary carbons. ^1^H and ^13^C NMR spectra of the **3** revealed signals of two trisubstituted double bonds (*δ*_H_ 5.13, d, *J* = 10.0 Hz; *δ*_C_ 124.5, CH, 141.2, C, and *δ*_H_ 5.11, m; *δ*_C_ 123.9, CH, 136.4, C), one trisubstituted epoxy group (*δ*_H_ 2.69, t, *J* = 3.6 Hz; *δ*_C_ 61.8, CH; 59.9, C) and three olefinic methyl groups (*δ*_H_ 1.85, s; *δ*_C_ 15.7; *δ*_H_ 1.73, s; *δ*_C_ 10.2 and *δ*_H_ 1.60, s; *δ*_C_ 15.1, respectively). The upfield ^13^C chemical shift of the olefinic methyl groups (*δ*_H_ 15.7 and 15.1) indicated the *E* geometry of the two trisubstituted double bonds. Further, a downfield signal at *δ*_H_ 8.47 (1H, brs) suggested the presence of a hydroperoxy group. The gross structure of **3** was established from the COSY and HMBC and was found to possess a dihydrofuranocembranoid skeleton close to sarcophytonin F (**9**) [16].

The relative configurations of the stereogenic centers of **3** were determined by inspection of NOE correlations, which revealed that the configuration of **3** was close to **9**, except that **3** possessed a weak NOE correlation between H-2 and H-16, which was not found in the NOESY spectrum of **9**. Further, it was found that **3** showed a W coupling signal of H-2 and H-16 (*J* = 3.0 Hz) and there are correlations between both protons in the COSY spectrum. On the basis of the above evidence, the orientation of H-16 in **3** was assigned to have the same *β*-orientation as H-2. On the other hand, **9** did not show any correlation between H-2 and H-16, demonstrating an *α*–orientation of H-16.

Sarcotenusene C (**4**), a colorless oil, had a pseudomolecular ion peak at m/z 339.1933 ${\left[M\;+\;\mathrm{Na}\right]}^{+}$ (calculated for C_20_H_28_O_3_Na: 339.1931) in the HRESIMS spectrum, indicating a molecular formula of C_20_H_28_O_3_. The IR spectrum revealed absorption bands at 3430, 1752, and 1661 cm^−1^, demonstrating hydroxy, carbonyl, and olefinic functional groups, respectively. The planar structure of **4** was determined by detailed analyses of the NMR spectroscopic data (Table 2 and Table 3) and COSY and HMBC correlations (Figure 2). Accordingly, the HMBC of H_3_-19 (*δ*_H_ 1.57, s) to C-7 (*δ*_C_ 124.5, CH), C-8 (*δ*_C_ 133.9, C), and C-9 (*δ*_C_ 38.4, CH_2_) suggested the presence of a trisubstituted double bond at C-7 and C-8. The more shielded ^13^C signals of two olefinic methyl groups (*δ*_C_ 16.5 and 16.4) indicated the *E* geometry of two trisubstituted double bonds. Thus, except for the C-4 configuration, the structure of **4** was determined.

It is worth mentioning that in this investigation, a rare hydroquinone monoterpenoid of 3,5,6-trimethyl-1,4-dihydroxybenzene substituted with a rare monoterpenoid side chain was discovered. To explore natural marine products as new drug leads, the anti-inflammatory activity, cytotoxicity, and PPAR-γ transcription factor assay of all the isolated metabolites were examined. The results demonstrated that all compounds were inactive in the PPAR-γ transcription factor assay, while anti-inflammatory assays revealed that (+)-7*α*,8*β*-dihydroxydeepoxysarcophine (13) potentially inhibited IL-1*β* production to 56 ± 1% in LPS-stimulated murine macrophage J774A.1 cells at a concentration of 30 μm. A previous study showed that a derivative of 1, flexibiliquinone, potentially inhibited the production of iNOS and COX-2 proteins in LPS-stimulated RAW264.7 macrophage cells [30]; however, in this study, **1** did not inhibit the production of pro-inflammatory cytokines such as TNF-*α* and IL-1*β* in LPS-stimulated J774A.1 macrophage cells. Importantly, **1** showed cytotoxicity towards breast cancer cell lines MCF-7 and MDA-MB-231 with IC_50_ values of 25.3 ± 2.8 and 36.4 ± 3.6 *µ*M, respectively. In addition, **2** was the only compound to exhibit cytotoxicity against the MCF-7 cell line with an IC_50_ value of 34.3 ± 3.7 *µ*M. Furthermore, dihydrofuranocembranoids **10** and **11** that exhibited in vitro cytotoxicity against MCF-7 and HepG2 cancer cell lines were ascertained. The results for active compounds are shown in Table 4.

**Figure 4:**
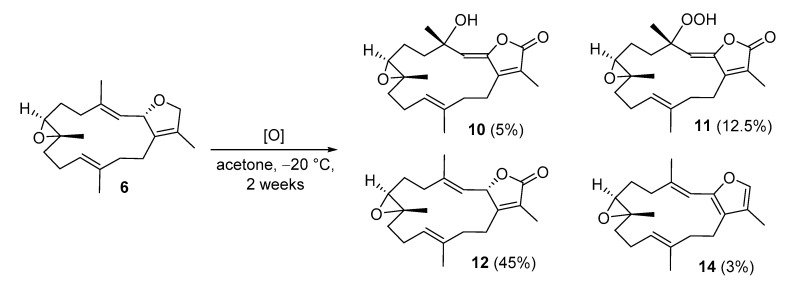
Autoxidation of 2*S*, 7*S*, 8*S*-sarcophytoxide (**6**) and the yield of final products.

The autoxidation of 2*S*, 7*S*, 8*S*-sarcophytoxide (**6**) was examined by storing acetone solution of 6 at −20 °C for two weeks. It was found that the amount of **6** was decreased; on the contrary, the conversion of **6** into **10**, **11**, **12**, and **14** was found in this study (Figure 4). This result was compatible with the previous work by Kobayashi et al. in 1991 [20]. Moreover, our investigation led to the isolation of 2*S*, 7*R*, 8*R*-sarcophytoxide (**7**) in a relatively smaller quantity than **6** in *S. tenuispiculatum*. On the basis of this result, it was expected that the massive extraction of *S. tenuispiculatum* can lead to the isolation of more diversified dihydrofuranocembranoids.

## 3. Experimental Section

### 3.1. General Experimental Procedures

Values of optical rotations of all the compounds were measured on a JASCO P-1020 polarimeter (JASCO Corporation, Tokyo, Japan). IR spectra were recorded on an FT/IR-4100 infrared spectrophotometer (JASCO Corporation, Tokyo, Japan). NMR spectra were recorded on a Varian 300 and 400 MR FT-NMR and Varian Unity INOVA500 FT-NMR (Varian Inc., Palo Alto, CA, USA) instruments at 300 MHz, 400 MHz, and 500 MHz for ^1^H and 100 MHz and 125 MHz for ^13^C in CDCl_3_ at 25 °C. Low-resolution electrospray ionization mass spectrometry (LRESIMS) and HRESIMS spectra were recorded on a Bruker APEX II (Bruker, Nremen, Germany) mass spectrometer. Silica gel (Merck, 230–400 mesh) was used for normal-phase column chromatography. Pre-coated silica gel plates (Merck, Kieselgel 60 F-254, 0.2 mm, Merck, Darmstadt, Germany) were used for analytical thin layer chromatography (TLC). High-performance liquid chromatography was performed using a Hitachi L-2455 HPLC apparatus with a Supelco C18 column (250 × 21.2 mm, 5 μm; Supelco, Bellefonte, PA, USA).

### 3.2. Animal Material

**Figure 5:**
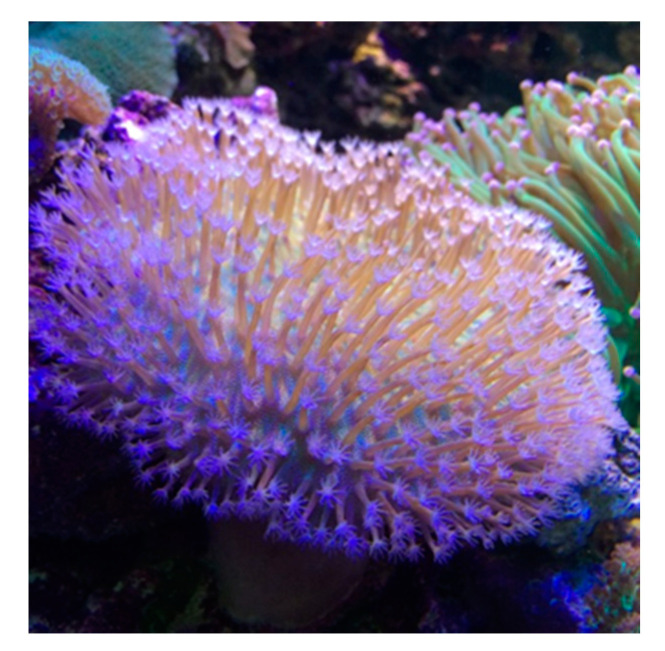
The soft coral *S. tenuispiculatum* cultured in an aquarium.

The soft coral *S. tenuispiculatum* was collected from southern Taiwan in 2013 and transplanted in a 90 × 60 × 45 cm^3^ aquarium with artificial coral reefs (Figure 5) at the Graduate Institute of Natural Products, Kaohsiung Medical University. The salinity of the seawater was controlled at 36 psu, and the temperature was maintained between 24 and 26 °C by a cooling system. Illumagic ComboRay CR-60 LED lighting was utilized to simulate sunshine and a TMS-SK-160 foam fractionator was used to remove waste particles. This organism was harvested in January 2018 and was stored in a freezer until extraction. For this study, a specimen was collected from the culture tank and sliced immediately in January 2018. The species was identified by Tzu–Hsuan Tu of the Department of Oceanography, National Sun Yat-sen University.

### 3.3. Extraction and Isolation

Animal sample of *S. tenuispiculatum* (wet weight: 180 g) was sliced followed by extraction with acetone (1 L × 3). The resulted oily residue was partitioned with ethyl acetate and H_2_O. The ethyl acetate soluble fraction (0.902 g) was purified over silica gel by column chromatography with elution of EtOAc in n-hexane (10−100%, stepwise) and then with MeOH in EtOAc (0−100%, stepwise) to yield 10 fractions. Fraction 3 (44.2 mg) eluted with n-hexane–EtOAc (7:1) was further purified by reversed-phase HPLC with acetonitrile–H_2_O (3:1) to yield 1 (3.5 mg). Fraction 2 was eluted with n-hexane–EtOAc (8:1) to obtain **6** subfractions (A–F). Subfraction 2A (17.3 mg) was further purified by reversed-phase HPLC with MeOH–H_2_O (8:1) to obtain **2** (2.0 mg) and **8** (6.3 mg). Subfraction 2B (77.2 mg) was also purified by reversed-phase HPLC with acetonitrile–H_2_O (5:1) to obtain **6** (17.5 mg), **7** (3.0 mg), **12** (23.5 mg), **13** (1.1 mg), and **14** (0.9 mg). Subfraction 2C (12.7 mg) was purified by reversed-phase HPLC with acetonitrile–H_2_O (4:1) to obtain **3** (3.4 mg) and **9** (4.5 mg) and subfraction 2D (9.8 mg) was purified by reversed-phase HPLC to obtain **4** (0.8 mg), **10** (1.2 mg), and **11** (3.2 mg). Furthermore, fraction 1 (33.2 mg) eluted with n-hexane–EtOAc (9:1) was further purified over silica gel with n-hexane–acetone (7:1) to yield **5** (13.1 mg).

Sarcotenuhydroquinone (**1**): colorless amorphous, ${\left[\alpha \right]}_{D}^{23}$ +15.5 (c 0.033, CHCl_3_), IR (KBr) v_max_ 3434, 2936, 2857, 1726, 1647, 1451, 1376, 1254, 1161, 1082, 636 cm^–1^; for ^13^C and ^1^H data see Table 1; electrospray ionization mass spectrometry (ESIMS) *m/z* 375; HRESIMS 375.2149 ${\left[M\;+\;\mathrm{Na}\right]}^{+}$ (calculated for C_20_H_32_O_5_Na: 375.2142).

Sarcotenusene A (**2**): colorless amorphous, ${\left[\alpha \right]}_{D}^{23}$ +11.3 (c 0.027, CHCl_3_), IR (KBr) v_max_ 3399, 2970, 2927, 1639, 1451, 1372, 794, 719 cm^–1^; for ^13^C and ^1^H data see Table 2 and Table 3; ESIMS *m/z* 305; HRESIMS 305.2474 ${\left[M\;+\;H\right]}^{+}$ (calculated for C_20_H_33_O_2_: 305.2475).

Sarcotenusene B (**3**): colorless amorphous, ${\left[\alpha \right]}_{D}^{23}$ +80.9 (c 0.033, CHCl_3_), IR (KBr) v_max_ 3452, 2922, 2860, 1749, 1670, 1451, 1376, 1013 cm^–1^; for ^13^C and ^1^H data see Table 2 and Table 3; ESIMS *m/z* 357; HRESIMS 357.2032 ${\left[M\;+\;\mathrm{Na}\right]}^{+}$ (calculated for C_20_H_30_O_4_Na: 357.2036).

Sarcotenusene C (**4**): colorless amorphous, ${\left[\alpha \right]}_{D}^{23}$ +21.8 (c 0.08, CHCl_3_), IR (KBr) v_max_ 3430, 2927, 2857, 1752, 1661, 1451, 1372, 1065, 758 cm^–1^; for ^13^C and ^1^H data see Table 2 and Table 3; ESIMS *m/z* 339; HRESIMS 339.1933 ${\left[M\;+\;\mathrm{Na}\right]}^{+}$ (calculated for C_20_H_28_O_3_Na: 339.1931).

### 3.4. Cytotoxicity Testing

Cell lines were purchased from the American Type Culture Collection (Rockville, MD, USA). Cytotoxicity testing for all compounds **1**–**14** was carried out using 3-(4,5-dimethylthiazol-2-yl)-2,5-diphenyltetrazolium bromide (MTT) assay as in the previous report [31,32]. Doxorubicin, the positive control in the MTT assay, showed cytotoxicity towards MCF-7, MDA-MB-231, HepG2, and HeLa cancer cell lines, with IC_50_ values of 6.8 ± 1.4, 6.3 ± 1.2, 9.6 ± 1.8, and 8.1 ± 2.1 (*µ*M), respectively. The compound was regarded as inactive when IC_50_ > 40 *µ*M.

### 3.5. Anti-Inflammatory Assay

Murine macrophage cell line J774A.1 was purchased from the American Type Culture Collection. Screening about the generation of pro-inflammatory cytokines in LPS-stimulated J774A.1 macrophage cells was carried out according to the previous report [32]. The concentrations of the produced cytokines were measured using ELISA according to the previous work [33] and manufacturer′s protocol.

### 3.6. PPAR-γ Transcription Factor Assay

The PPAR-γ transcription factor assay of all the compounds was carried out as described previously [34].

## 4. Conclusions

Marine soft corals have been regarded as an important source of bioactive secondary metabolites [35,36,37]. Chemical study of *S. tenuispiculatum* led to the isolation of one new monoterpenoidal hydroquinone **1** and three new cembranoids **2**−**4**, along with ten known cembranoids **5**−**14**. Compound **1** was found to exhibit significant cytotoxicity towards human breast cancer cell lines MCF-7 and MDA-MB-231. Cembranoids **2**, **6**, **7**, **9**, **10**, and **11** also displayed various cytotoxicity towards a limited panel of cancer cells. In addition, at a concentration of 30 *µ*M, **13** could downregulate the production of IL-1*β* to 56 ± 1% in LPS-stimulated J774A.1 macrophage cells. On the basis of the above evidence, we suggest that *S. tenuispiculatum* is a good source of natural bioactive products.

## Figures and Tables

**Table 1:** ^13^C, ^1^H, correlation spectroscopy (COSY), and heteronuclear multiple bond correlation (HMBC) spectroscopic data of **1**.

Position	1			
	^13^C*^α^*	^1^H ^b^	COSY	HMBC
1	177.3 (C)			
2	39.4 (CH)^c^	2.46, sext (6.8)^d^	H_2_-3, H_3_-9	C-1, C-3
3	34.2 (CH_2_)	1.41, m; 1.65 m	H_2_-2, H_2_-4	
4	21.3 (CH_2_)	1.42, m	H_2_-3, H_2_-5	
5	39.3 (CH_2_)	1.49, m; 1.56, m	H_2_-4	
6	74.2 (C)			
7	31.5 (CH_2_)	1.76, m	H_2_-8	C-6, C-8, C-1′
8	20.7 (CH_2_)	2.60, t (7.2)	H_2_-7	C-6, C-7, C-1′, C-2′, C-6′
9	17.0 (CH_3_)	1.14, d (7.2)		C-1, C-2, C-3
10	23.7 (CH_3_)	1.21, s		C-5, C-6, C-7
1′	117.2 (C)			
2′	118.5 (C)			
3′	144.6 (C)			
4′	121.0 (C)			
5′	122.6 (C)			
6′	145.3 (C)			
7′	11.3 (CH_3_)	2.11, s		C-1′, C-2′, C-3′
8′	12.2 (CH_3_)	2.16, s		C-3′, C-4′, C-5′
9′	11.8 (CH_3_)	2.10, s		C-4′, C-5′, C-6′
OMe	51.5 (CH_3_)	3.66, s		C-1

*^α^* The ^13^C spectroscopic data of **1** were recorded at 100 MHz in CDCl_3_. ^b^ The ^1^H spectroscopic data of **1** were recorded at 400 MHz in CDCl_3_. ^c^ Attached protons were deduced from the results of DEPT experiments. ^d^ *J* values (in Hz) in parentheses.

**Table 2:** ^13^C NMR spectroscopic data of **2**–**4**.

Position	2 ^α^	3 ^α^	4 ^b^
1	153.8 (C)	142.5 (C)	151.9 (C)
2	119.8 (CH) ^c^	82.6 (CH)	148.1 (C)
3	58.5 (CH)	124.5 (CH)	124.5 (CH)
4	61.9 (C)	141.2 (C)	72.9 (C)
5	36.8 (CH_2_)	37.6 (CH_2_)	42.5 (CH_2_)
6	21.8 (CH_2_)	25.2 (CH_2_)	24.5 (CH_2_)
7	125.4 (CH)	61.8 (CH)	124.5 (CH)
8	134.5 (C)	59.9 (C)	133.9 (C)
9	39.1 (CH_2_)	39.7 (CH_2_)	38.4 (CH_2_)
10	24.2 (CH_2_)	23.5 (CH_2_)	23.0 (CH_2_)
11	125.1 (CH)	123.9 (CH)	126.8 (CH)
12	135.1 (C)	136.4 (C)	131.5 (C)
13	40.8 (CH_2_)	36.5 (CH_2_)	36.7 (CH_2_)
14	28.0 (CH_2_)	26.3 (CH_2_)	22.7 (CH_2_)
15	73.7 (C)	124.4 (C)	123.2 (C)
16	29.9 (CH_3_)	114.3 (CH)	170.1 (C)
17	30.0 (CH_3_)	10.2 (CH_3_)	9.1 (CH_3_)
18	18.9 (CH_3_)	15.7 (CH_3_)	30.3 (CH_3_)
19	15.9 (CH_3_)	16.9 (CH_3_)	16.4 (CH_3_)
20	16.0 (CH_3_)	15.1 (CH_3_)	16.5 (CH_3_)

^α^ Spectroscopic data of **2** and **3** were recorded at 100 MHz in CDCl_3_. ^b^ Spectroscopic data of **4** were recorded at 125 MHz in CDCl_3_. ^c^ Attached protons were deduced from DEPT experiments.

**Table 3:** ^1^H NMR spectroscopic data of **2**–**4**.

Position	2 ^α^	3 ^α^	4 ^b^
2	5.37, d (7.6) ^c^	5.67, d (10.0)	
3	3.42, d (7.6)	5.13, d (10.0)	5.30, s
5	1.68, m	2.37, m	1.96, m
	1.99, m		
6	1.98, m	1.65, m	2.10, m
	2.15, m	1.94, m	
7	5.00, t (6.4)	2.69, t (3.6)	4.90, t (7.0)
9	2.01, m	1.01, t (12.8)	1.97, m
	2.12, m	2.12, dt (3.6, 12.8)	
10	2.12, m	1.91, m	2.12 m
	2.18, m	2.27 m	2.38 m
11	5.07, t (6.8)	5.11, m	4.92, t (7.0)
13	2.20, m	1.92, m	2.31, m
	2.23, m	2.00, m	
14	2.25, m	1.72, m	2.52 m
	2.50, m	2.59, m	2.57 m
16	1.37, s	5.97, d (4.0)	
17	1.37, s	1.73, s	1.94, s
18	1.28, s	1.85, s	1.45, s
19	1.57, s	1.28, s	1.57, s
20	1.64, s	1.60, s	1.58, s
OOH		8.47, brs	

^α^ Spectroscopic data of **2** and **3** were recorded at 400 MHz in CDCl_3_. ^b^ Spectroscopic data of **4** were recorded at 500 MHz in CDCl_3_. ^c^ *J* values (in Hz) in parentheses.

**Table 4:** Cytotoxicity (*µ*M) of compounds **1**–**14**.

Compound	MCF-7	MDA-MB-231	HepG2	HeLa
**1**	25.3 ± 2.8	36.4 ± 3.6	– ^a^	–
**2**	34.3 ± 3.7	–	–	–
**6**	37.6 ± 4.2	–	35.2 ± 4.4	–
**7**	33.3 ± 3.5	–	28.6 ± 3.4	–
**9**	30.1 ± 3.1	38.6 ± 5.0	–	–
**10**	24.3 ± 3.0	–	34.5 ± 4.2	–
**11**	27.2 ± 4.0	–	36.4 ± 5.3	–
Doxorubicin	6.8 ± 1.4	6.3 ± 1.2	9.6 ± 1.8	8.1 ± 2.1

^a^ > 40 *µ*M. Values are means ± SEM (n = 3).

## Data Availability

Data available in a publicly accessible repository.

## Supplementary Materials

The following ESIMS, HRESIMS, IR, 1H, 13C , DEPT , HSQC, COSY, HMBC, and NOESY NMR spectra of new compounds 1--4 are available online at https://www.mdpi.com/1660-3397/19/1/8/s1; Figure S1: ESIMS spectrum of 1; Figure S2: HRESIMS spectrum of 1; Figure S3: IR spectrum of 1; Figure S4: ^1^H NMR spectrum of 1 in CDCl_3_ at 400 MHz; Figure S5: ^1^H NMR spectrum (from 1.0 to 3.0 ppm) of 1 in CDCl_3_ at 400 MHz; Figure S6: ^13^C NMR spectrum of 1 in CDCl_3_ at 100 MHz; Figure S7: DEPT spectrum of 1; Figure S8: HSQC spectrum of 1; Figure S9: COSY spectrum of 1; Figure S10: HMBC spectrum of 1; Figure S11: NOESY spectrum of 1; Figure S12: ESIMS spectrum of 2; Figure S13: HRESIMS spectrum of 2; Figure S14: IR spectrum of 2; Figure S15: ^1^H NMR spectrum of 2 in CDCl_3_ at 400 MHz; Figure S16: ^1^H NMR spectrum (from 1.0 to 3.5 ppm) of 2 in CDCl_3_ at 400 MHz; Figure S17: ^13^C NMR spectrum of 2 in CDCl_3_ at 100 MHz; Figure S18: DEPT spectrum of 2; Figure S19: HSQC spectrum of 2; Figure S20: COSY spectrum of 2; Figure S21: HMBC spectrum of 2; Figure S22: NOESY spectrum of 2; Figure S23: ESIMS spectrum of 3; Figure S24: HRESIMS spectrum of 3; Figure S25: IR spectrum of 3; Figure S26: ^1^H NMR spectrum of 3 in CDCl_3_ at 300 MHz; Figure S27: ^1^H NMR spectrum of 3 in CDCl_3_ at 400 MHz; Figure S28: ^1^H NMR spectrum (from 0.5 to 3.0 ppm) of 3 in CDCl_3_ at 400 MHz; Figure S29: ^13^C NMR spectrum of 3 in CDCl_3_ at 100 MHz; Figure S30: DEPT spectrum of 3; Figure S31: HSQC spectrum of 3; Figure S32: COSY spectrum of 3; Figure S33: HMBC spectrum of 3; Figure S34: NOESY spectrum of 3; Figure S35: ESIMS spectrum of 4; Figure S36: HRESIMS spectrum of 4; Figure S37: IR spectrum of 4; Figure S38: ^1^H NMR spectrum of 4 in CDCl_3_ at 500 MHz; Figure S39: ^1^H NMR spectrum (from 1.2 to 3.0 ppm) of 4 in CDCl_3_ at 500 MHz; Figure S40: ^13^C NMR spectrum of 4 in CDCl_3_ at 125 MHz; Figure S41: DEPT spectrum of 4; Figure S42: HSQC spectrum of 4; Figure S43: COSY spectrum of 4; Figure S44: HMBC spectrum of 4; Figure S45: NOESY spectrum of 4.
