# Supplementary material for: New Hydroquinone Monoterpenoid and Cembranoid-Related Metabolites from the Soft Coral Sarcophyton tenuispiculatum
Source: Mar Drugs. 2020 Dec 27;19(1):8. doi: 10.3390/md19010008 (PMC7823492; doi:10.3390/md19010008)
Supplement: Supplementary file 1 [file marinedrugs-19-00008-s001.pdf]

New hydroquinone monoterpenoid and cembranoid-related metabolites from the soft coral *Sarcophyton tenuispiculatum*

Tzu-Yin Huang [1], Chiung-Yao Huang [2], Shu-Rong Chen [3], Jing-Ru Weng [1, 2], Tzu-Hsuan Tu [4], Yuan-Bin Cheng [2], Shih-Hsiung Wu [5] and Jyh-Horng Sheu [1,2,3,6,*]

[1] Doctoral Degree Program in Marine Biotechnology, National Sun Yat-sen University, Kaohsiung 804, Taiwan; HuangTY@g-mail.nsysu.edu.tw (T.-Y.H.); jrweng@mail.nsysu.edu.tw (J.-R.W.)
[2] Department of Marine Biotechnology and Resources, National Sun Yat-sen University, Kaohsiung 804, Taiwan; huangcy@mail.nsysu.edu.tw (C.-Y.H.); jmb@kmu.edu.tw (Y.-B.C.)
[3] Graduate Institute of Natural Products, College of Pharmacy, Kaohsiung Medical University, Kaohsiung 807, Taiwan; u106831002@kmu.edu.tw
[4] Department of Oceanography, National Sun Yat-sen University, Kaohsiung 804, Taiwan; thtu@mail.nsysu.edu.tw
[5] Institute of Biological Chemistry, Academia Sinica, Taipei 11529, Taiwan; shwu@gate.sinica.edu.tw
[6] Department of MedicalResearch, China Medical University Hospital, China Medical University, Taichung 404, Taiwan
* Correspondence: sheu@mail.nsysu.edu.tw; Tel.: +886-7-525-2000 (ext. 5030); Fax: +886-7-525-5020

Compound **1** spectroscopic data

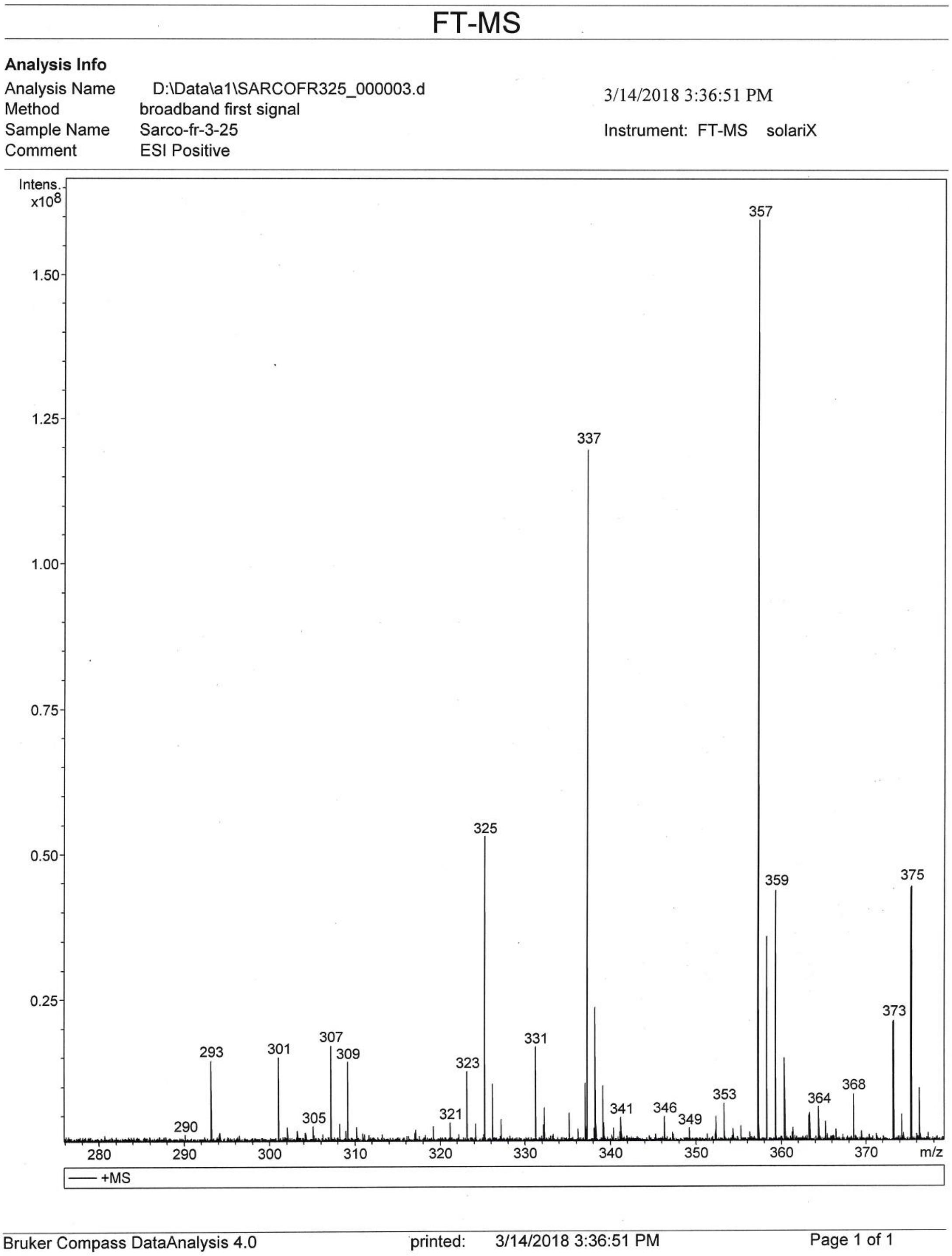

Figure S1: ESIMS spectrum of **1**

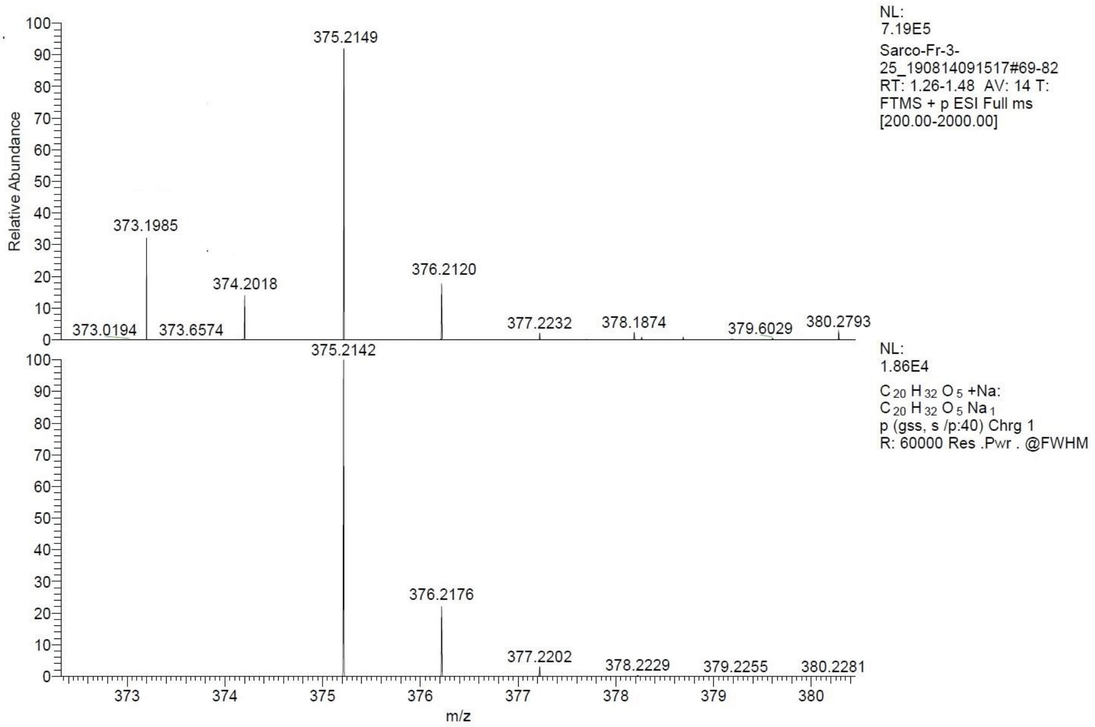

Figure S2: HRESIMS spectrum of **1**

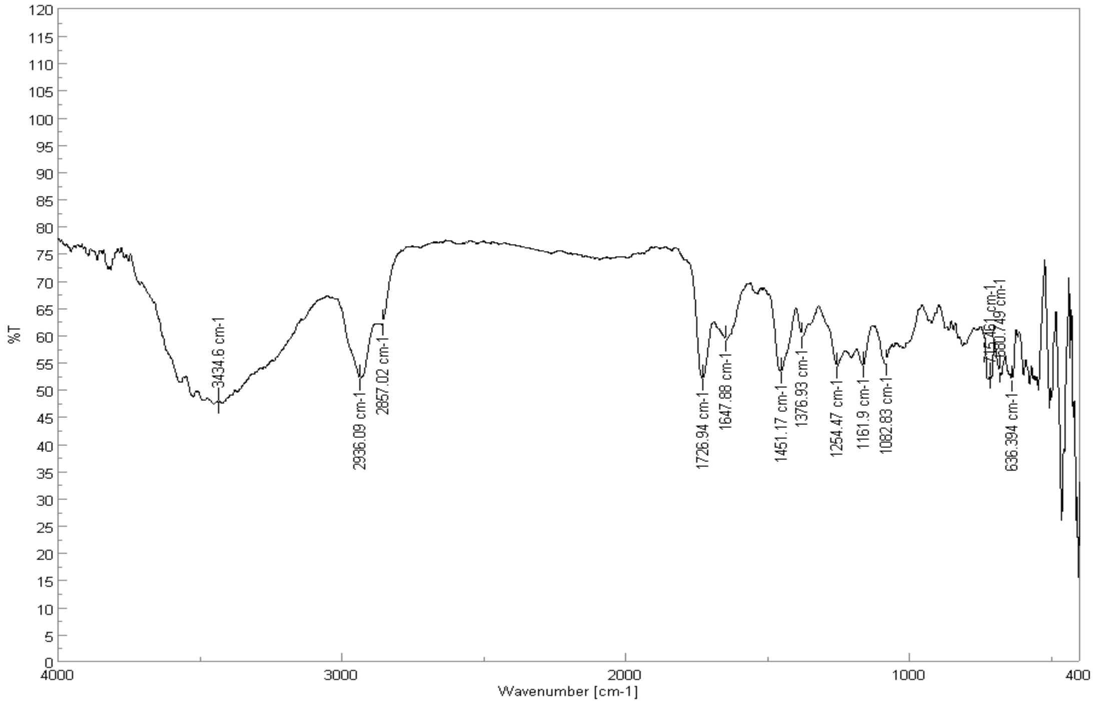

Figure S3: IR spectrum of **1**

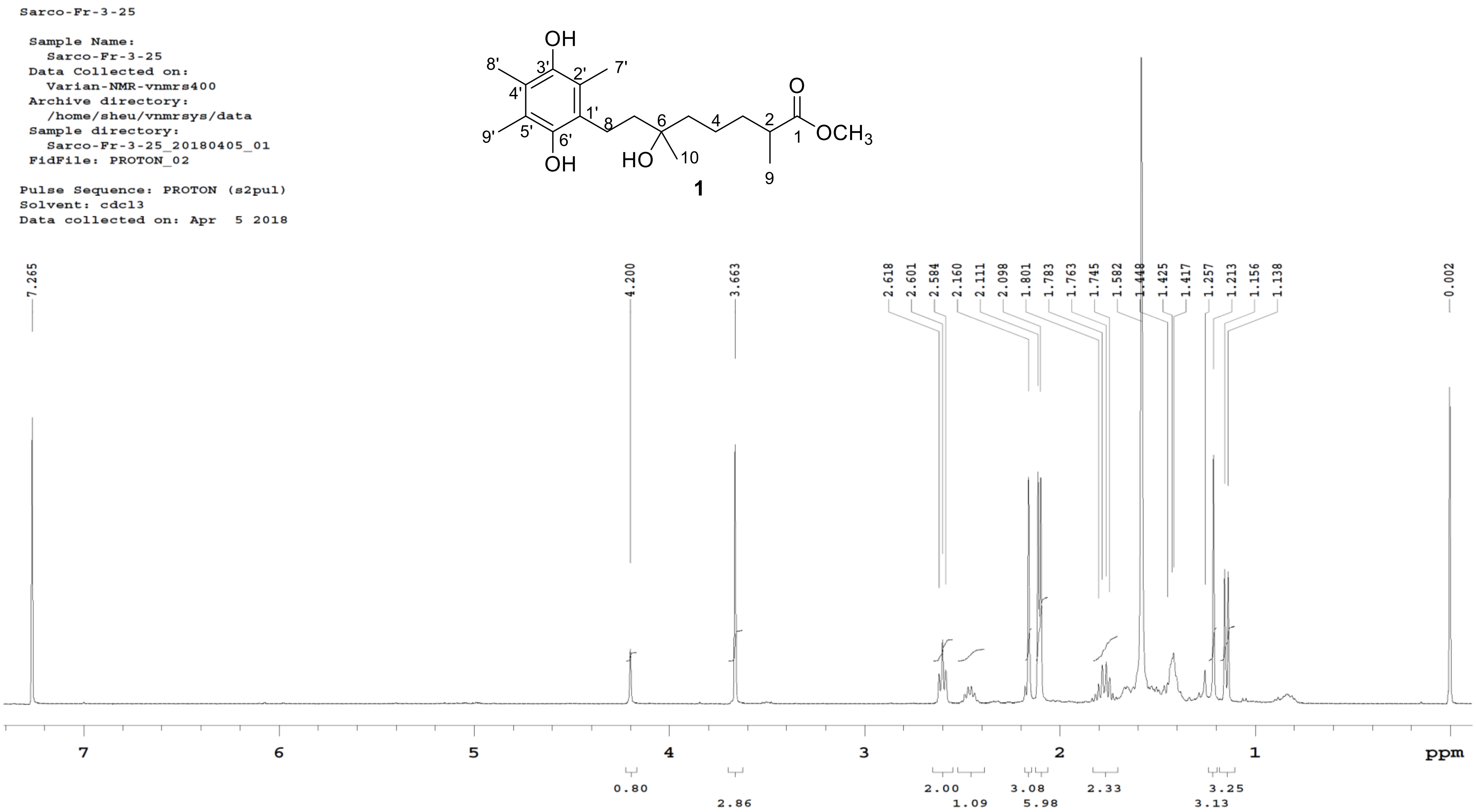

Figure S4: ^{1}H NMR spectrum of **1** in $CDCl_3$ at 400 MHz

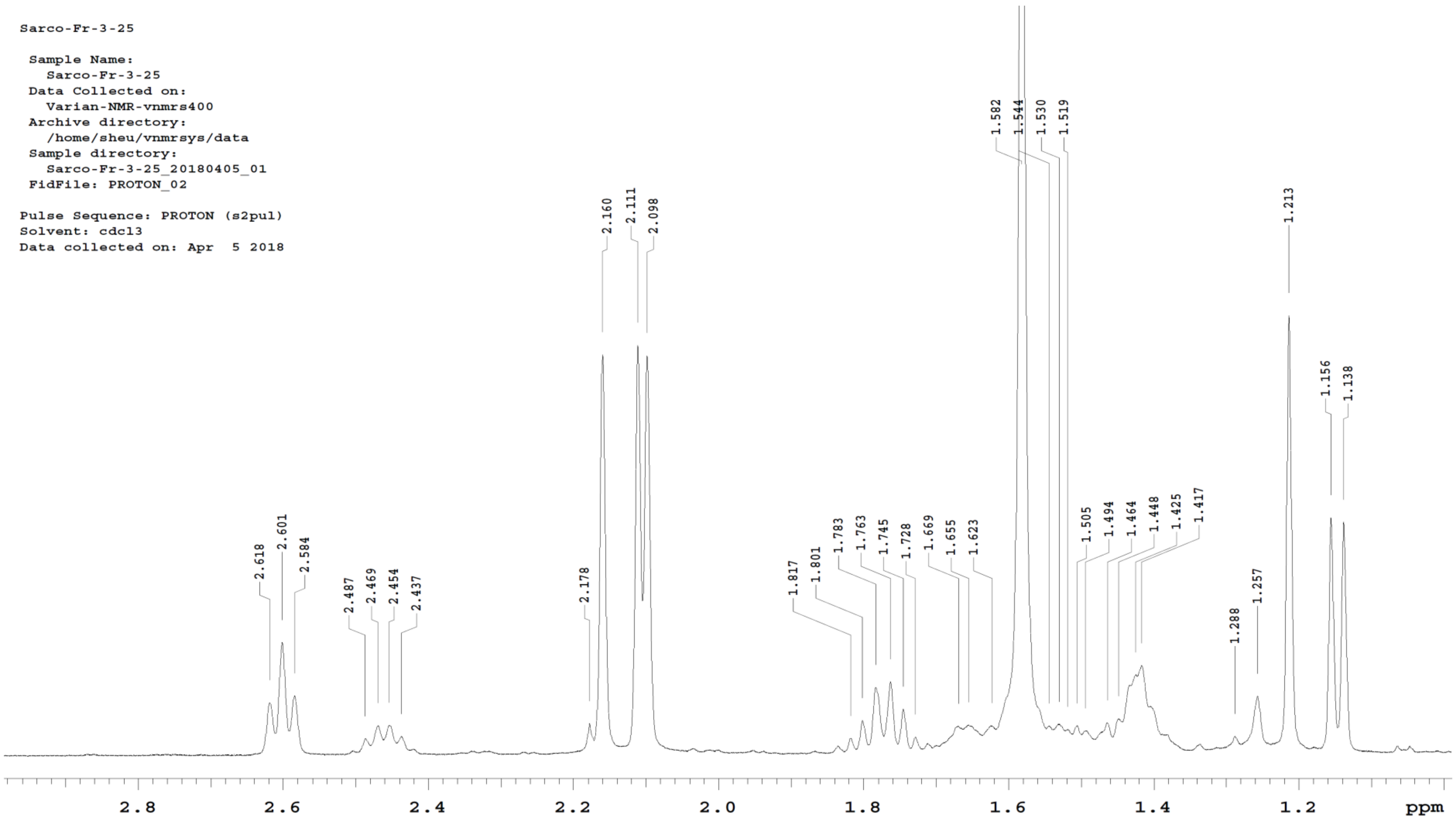

Figure S5: ^{1}H NMR spectrum (from 1.0 to 3.0 ppm) of **1** in $CDCl_3$ at 400 MHz

Figure S6: ^{13}C NMR spectrum of **1** in $CDCl_3$ at 100 MHz

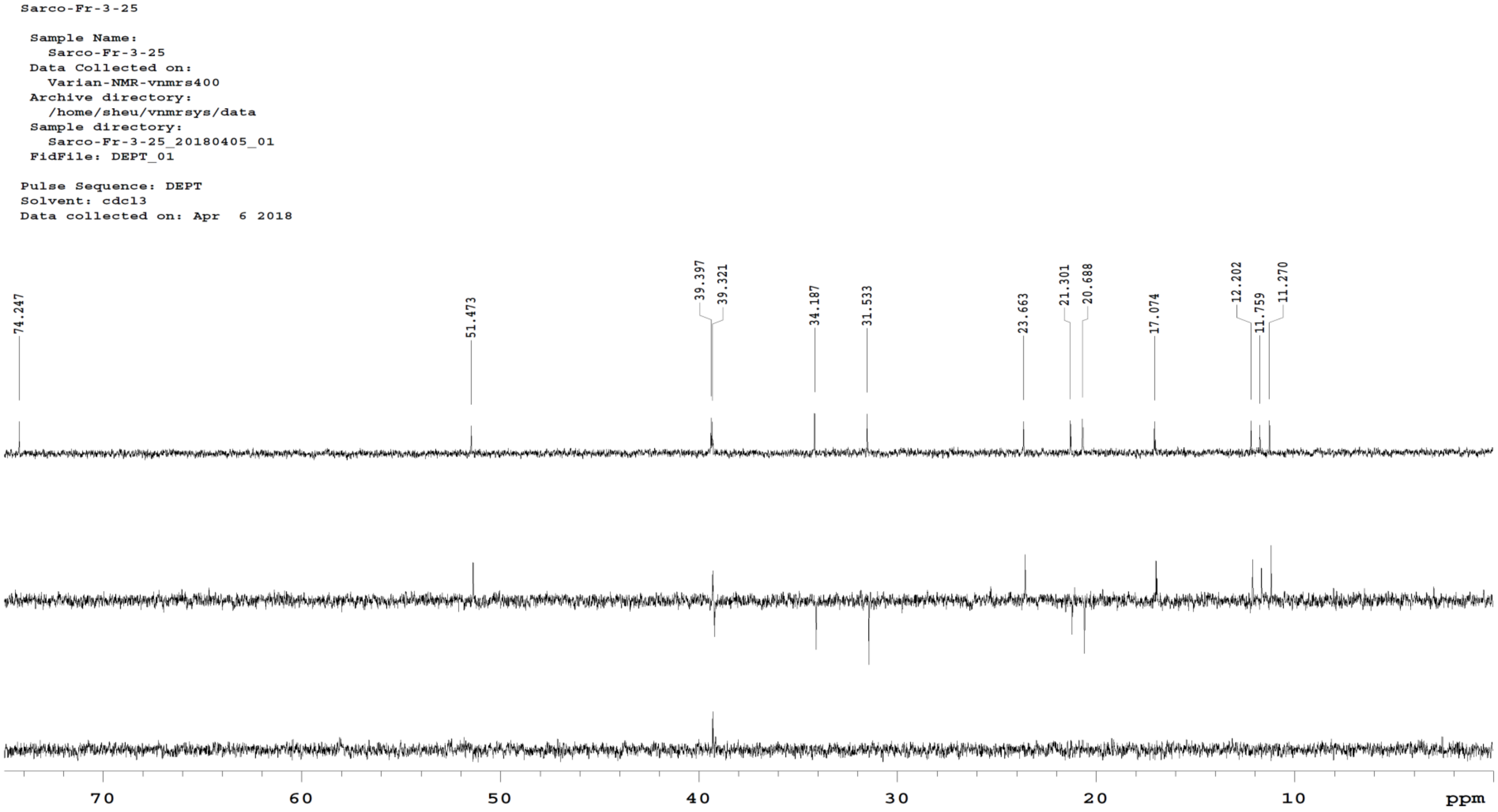

Figure S7: DEPT spectrum of **1**

Sarco-Fr-3-25

Sample Name:
Sarco-Fr-3-25
Data Collected on:
Varian-NMR-vnmrs400
Archive directory:
/home/sheu/vnmrsys/data
Sample directory:
Sarco-Fr-3-25_20180405_01
FidFile: HSQCAD_01

Pulse Sequence: HSQCAD
Solvent: cdcl3
Data collected on: Apr 5 2018

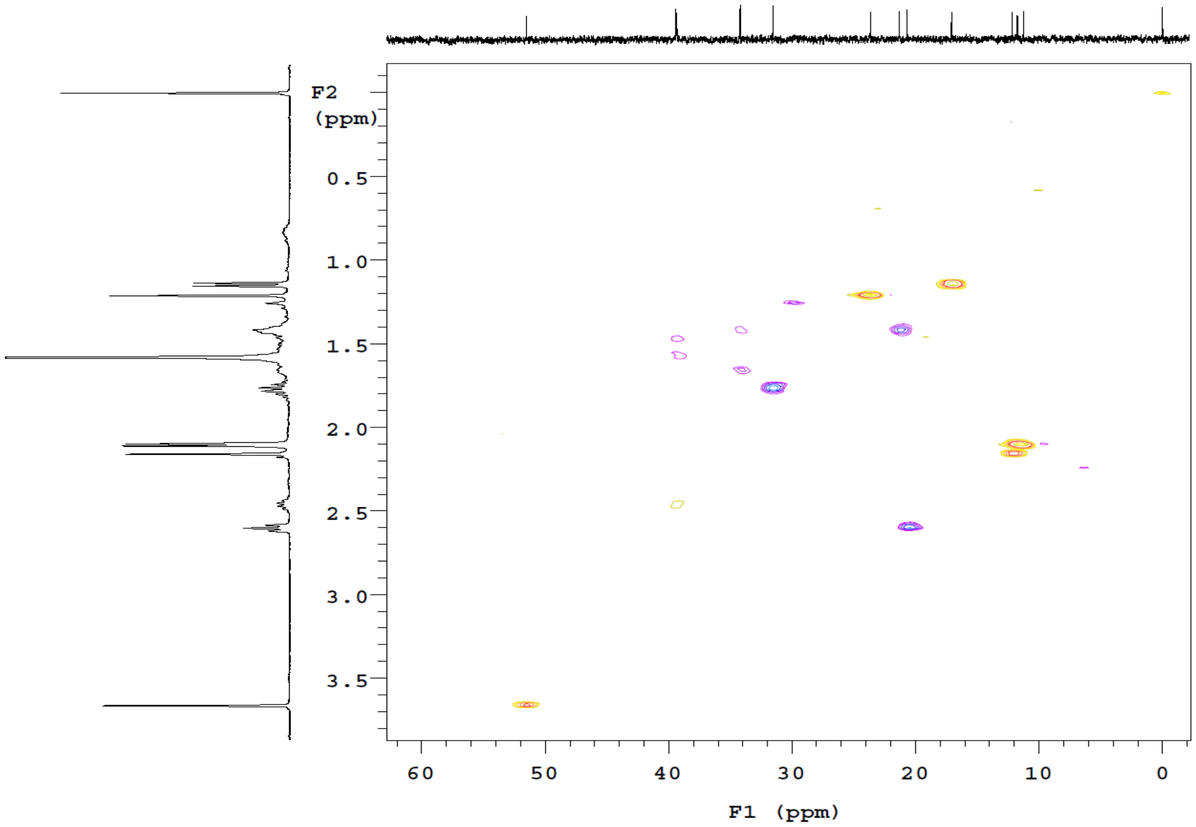

Figure S8: HSQC spectrum of **1**

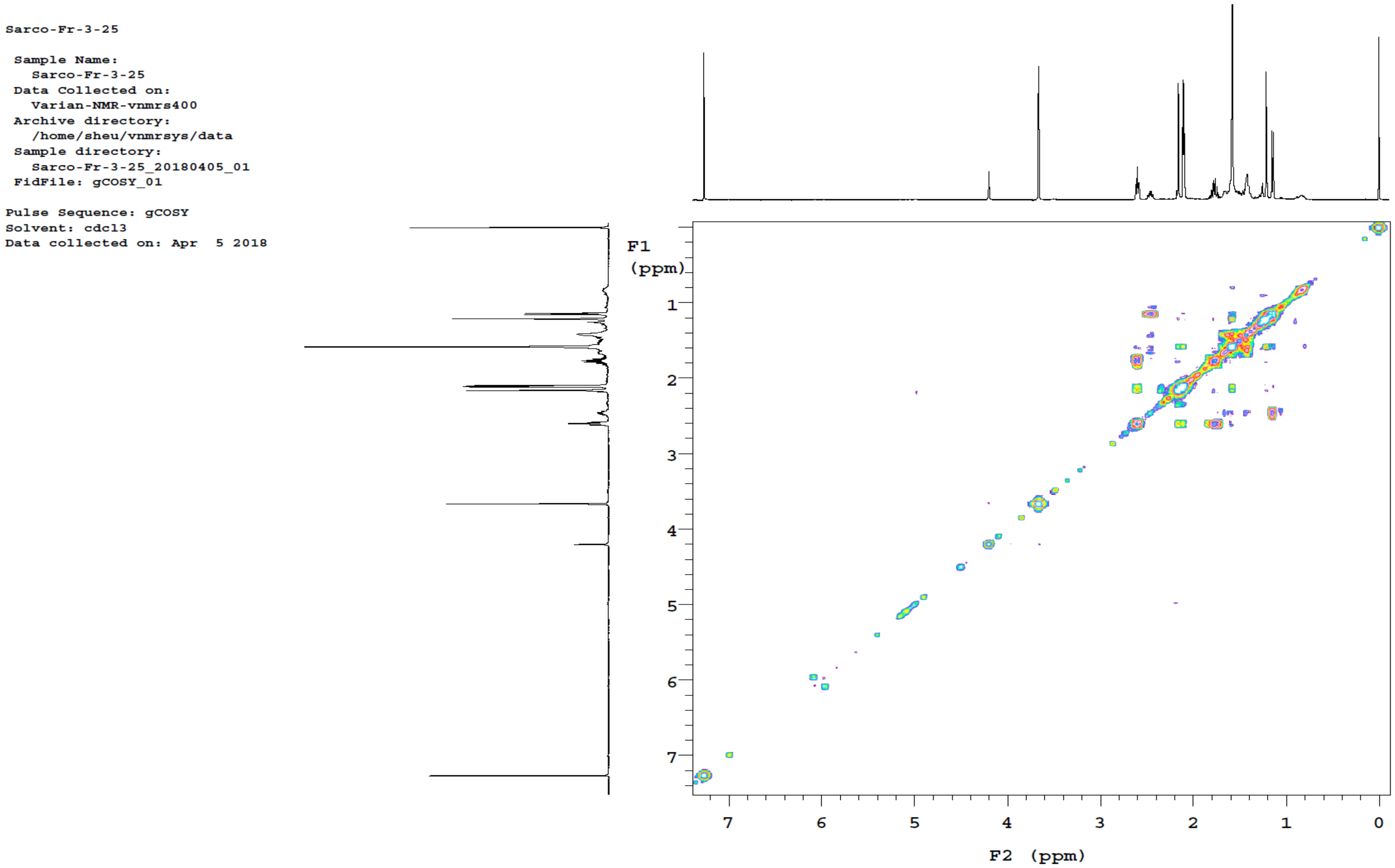

Figure S9: COSY spectrum of **1**

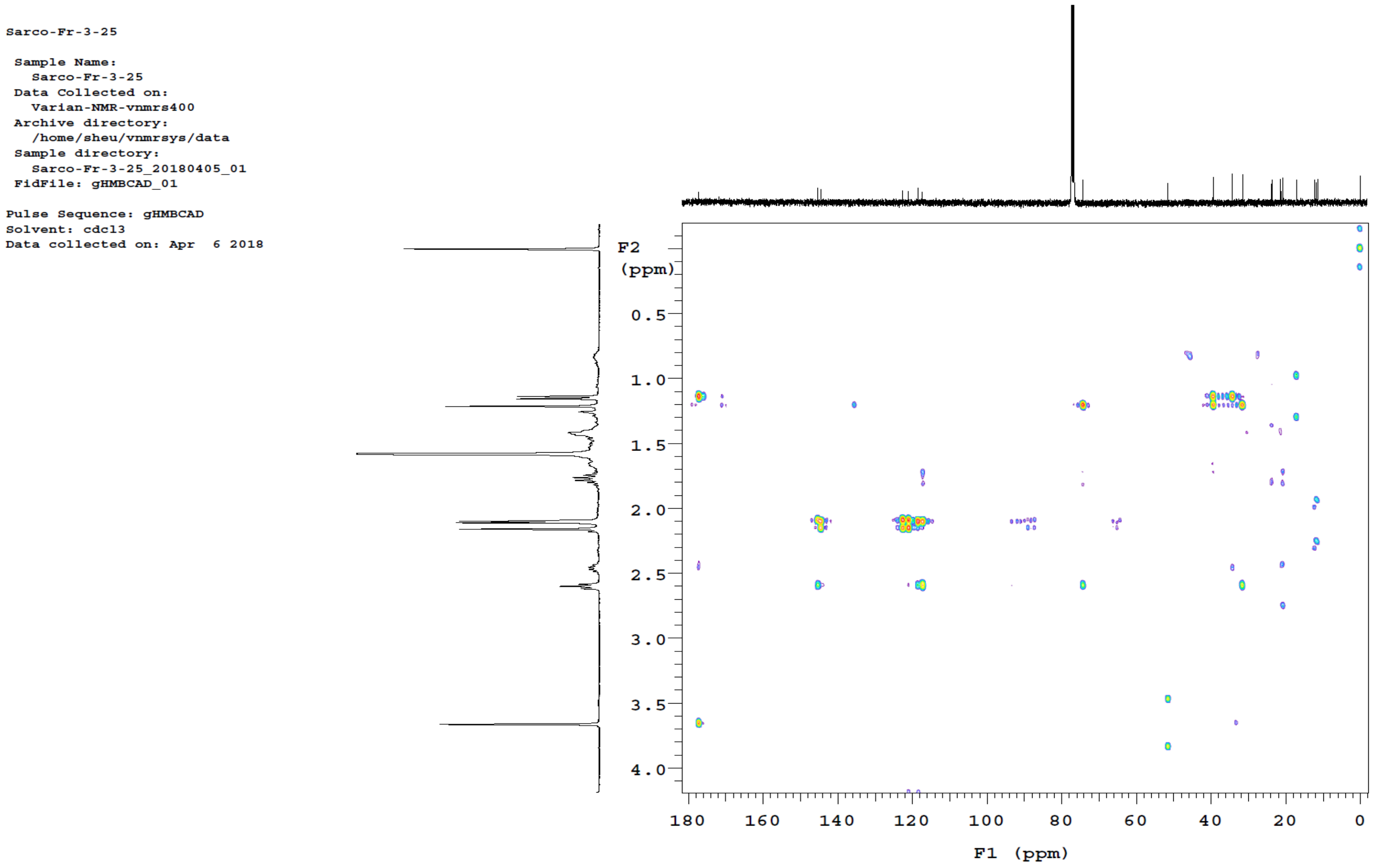

Figure S10: HMBC spectrum of **1**

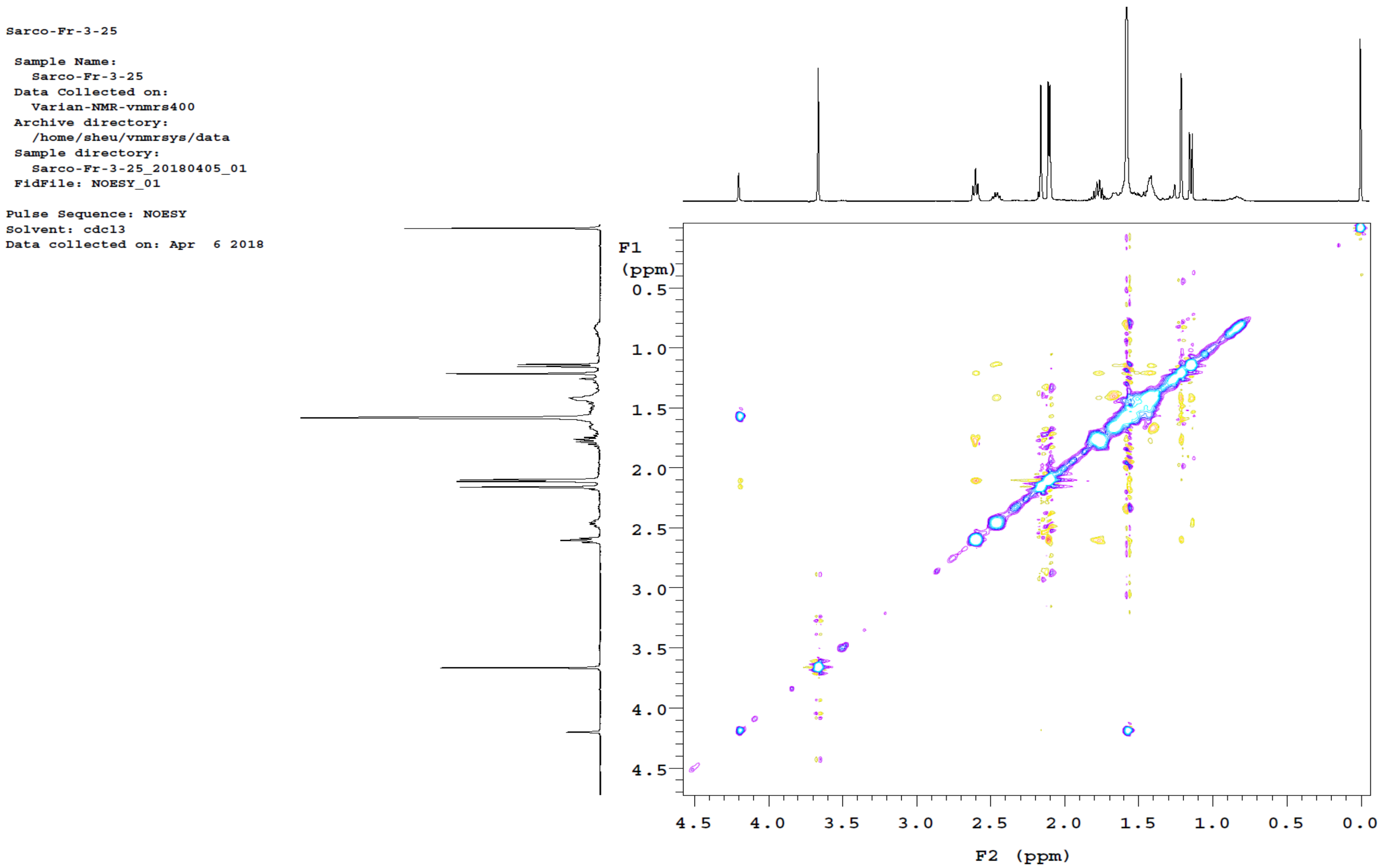

Figure S11: NOESY spectrum of **1**

Compound **2** spectroscopic data

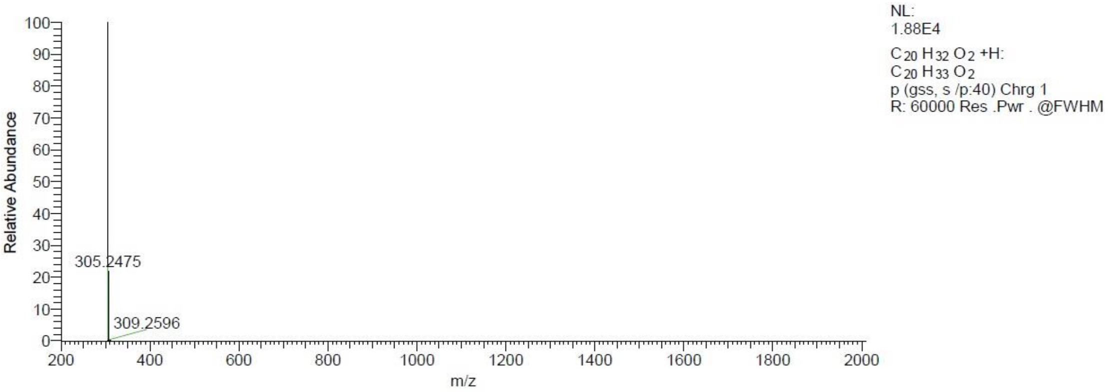

Figure S12: ESIMS spectrum of **2**

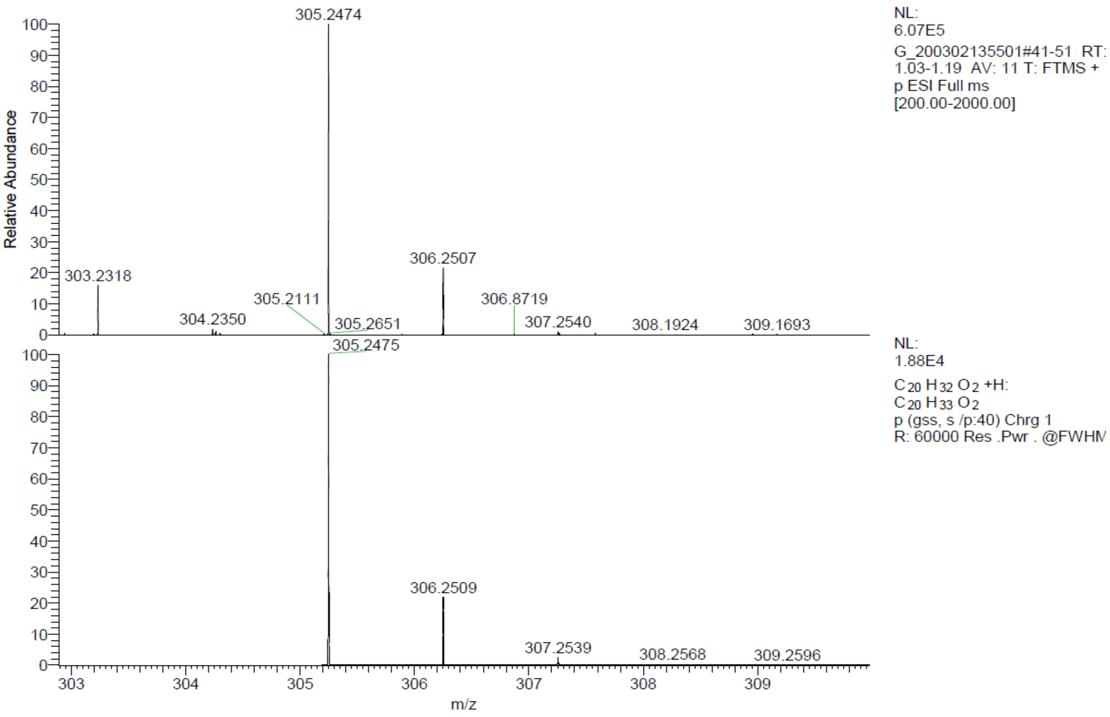

Figure S13: HRESIMS spectrum of **2**

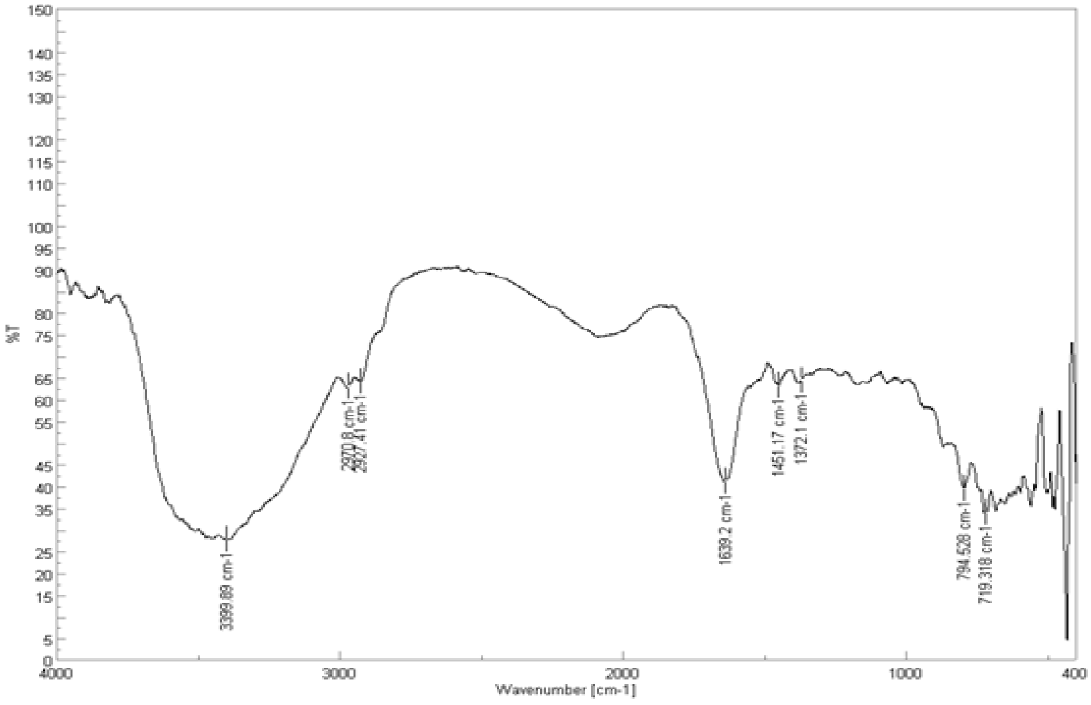

Figure S14: IR spectrum of **2**

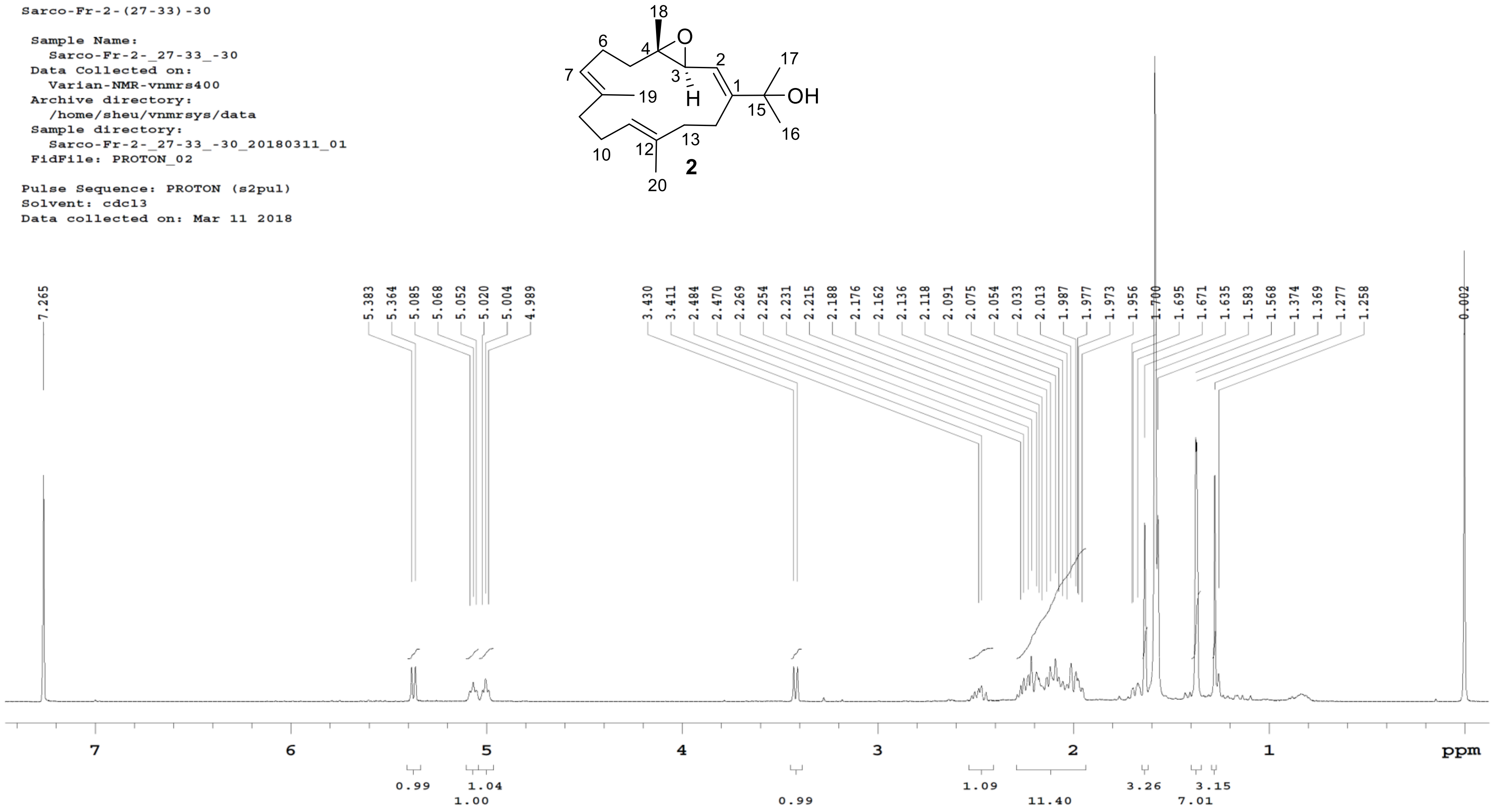

Figure S15: ^{1}H NMR spectrum of **2** in $CDCl_3$ at 400 MHz

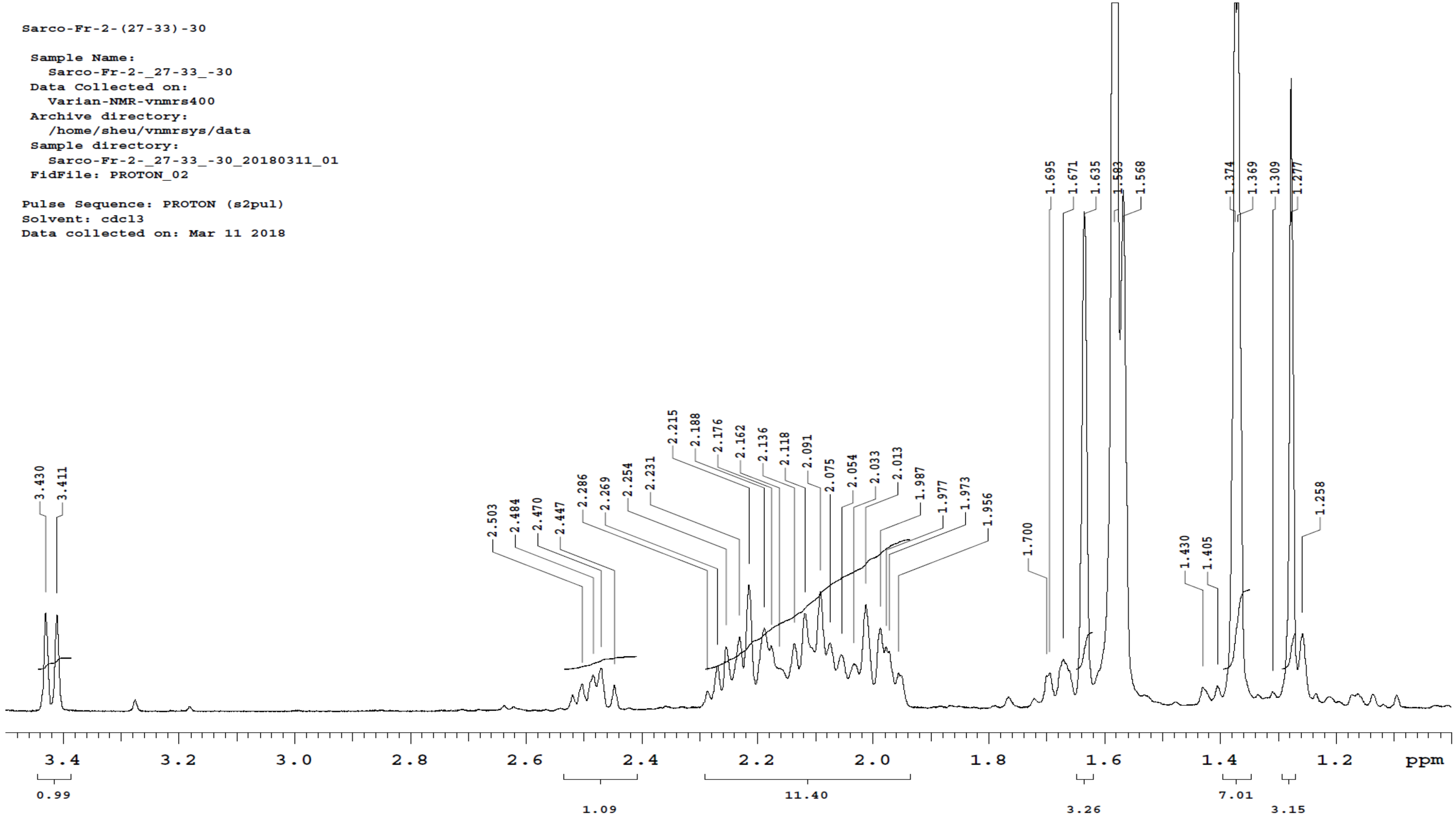

Figure S16: ^{1}H NMR spectrum (from 1.0 to 3.5 ppm) of **2** in $CDCl_3$ at 400 MHz

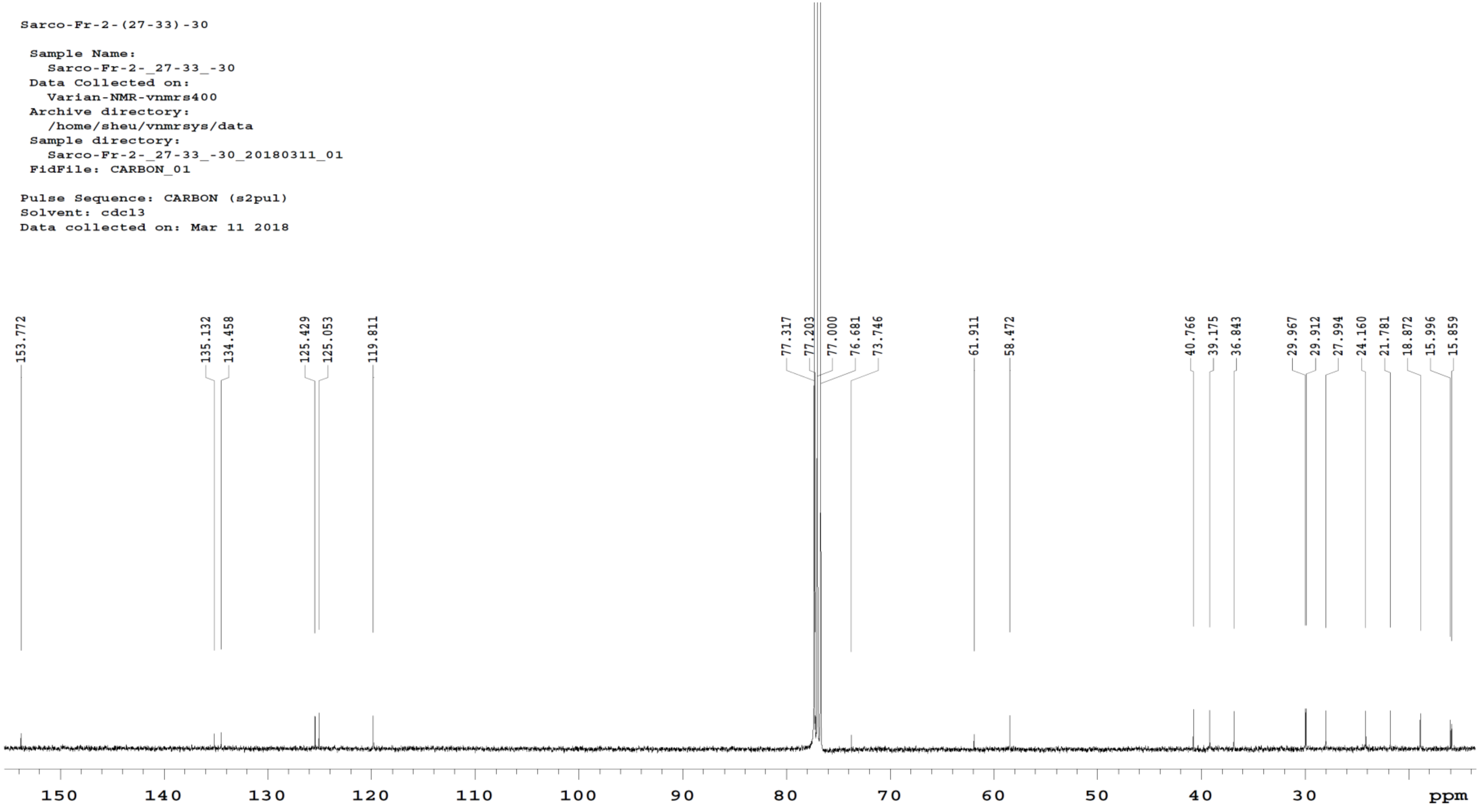

Figure S17: ^{13}C NMR spectrum of **2** in $CDCl_3$ at 100 MHz

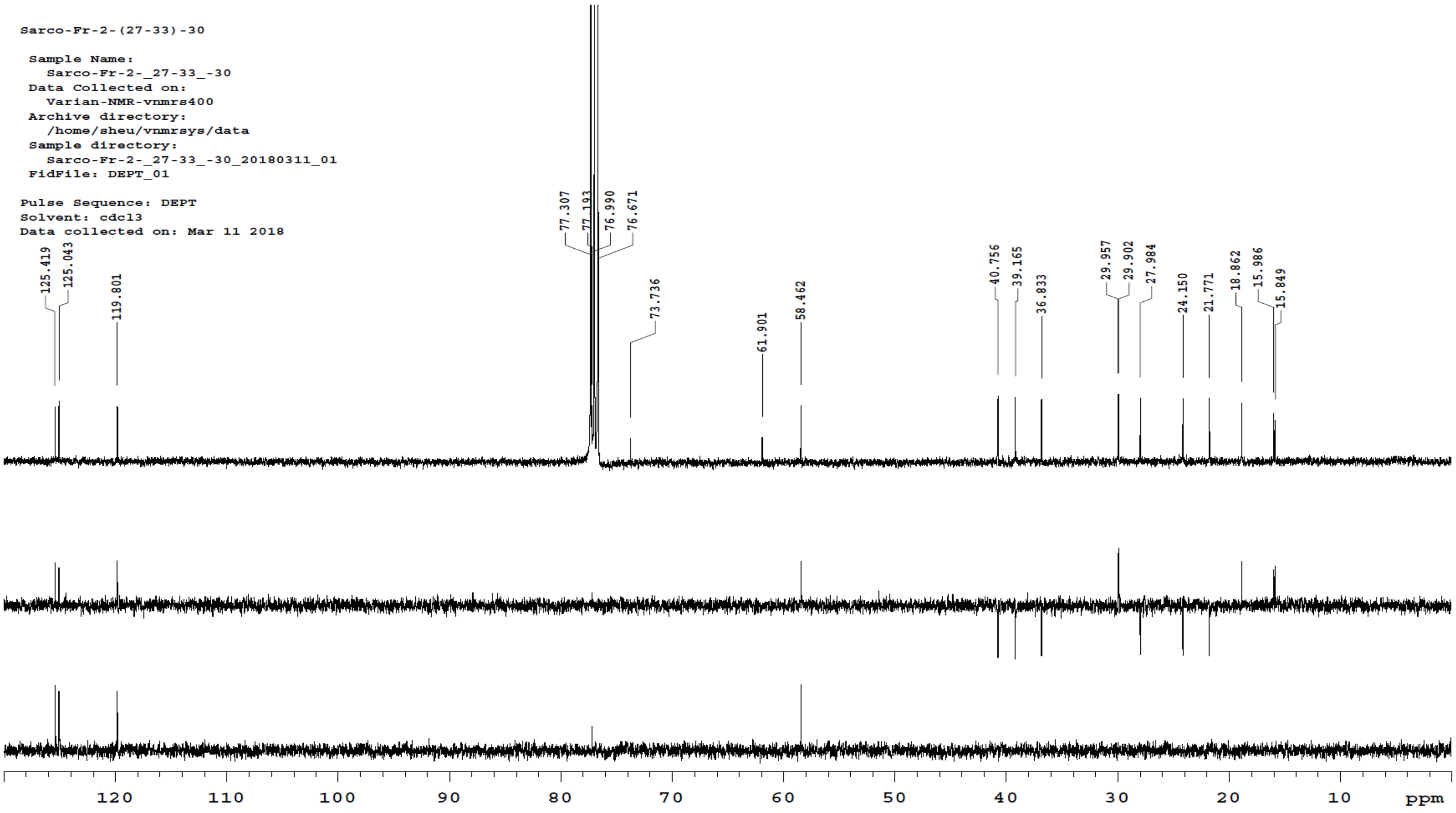

Figure S16: DEPT spectrum of **2**

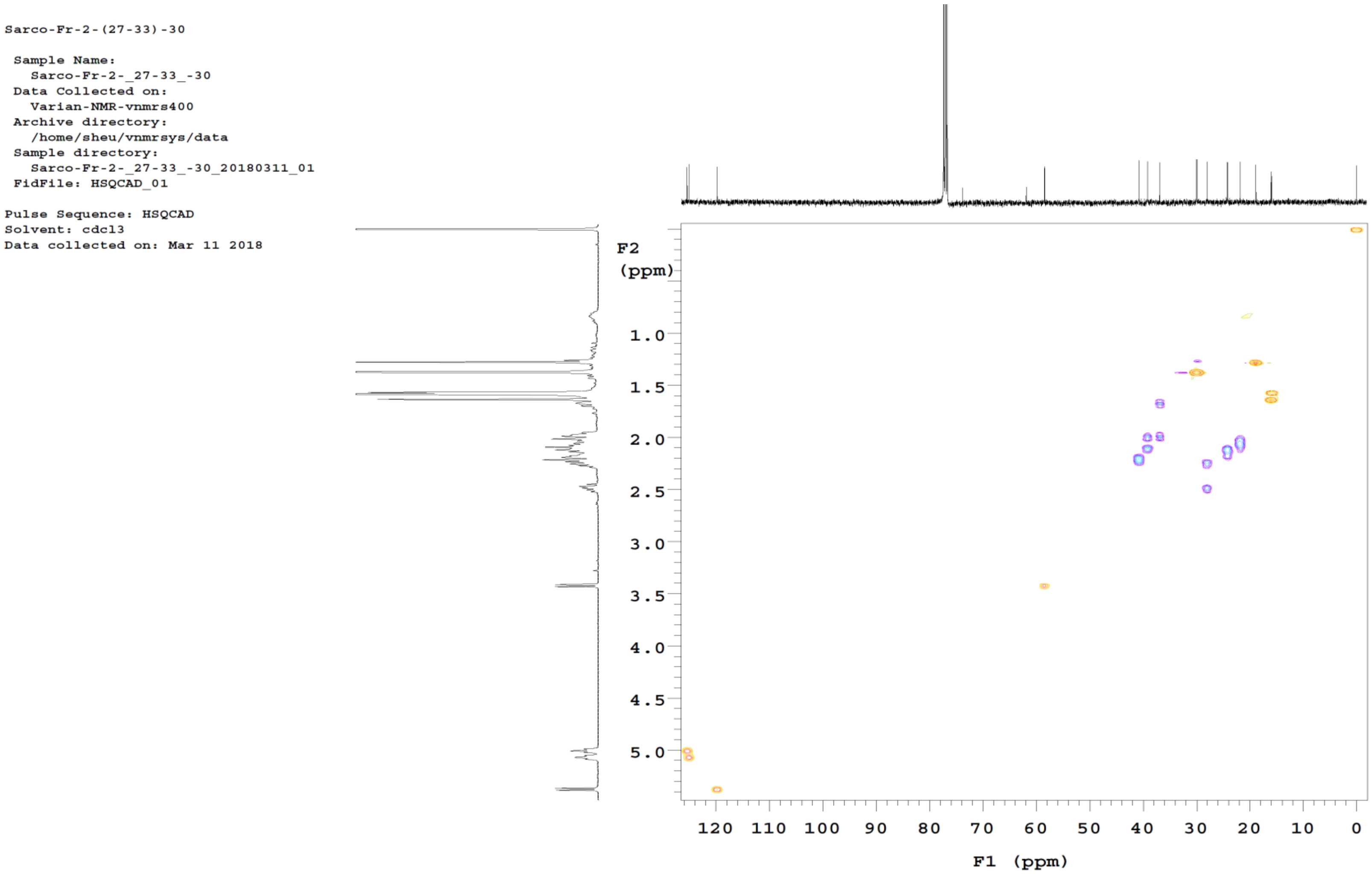

Figure S19: HSQC spectrum of **2**

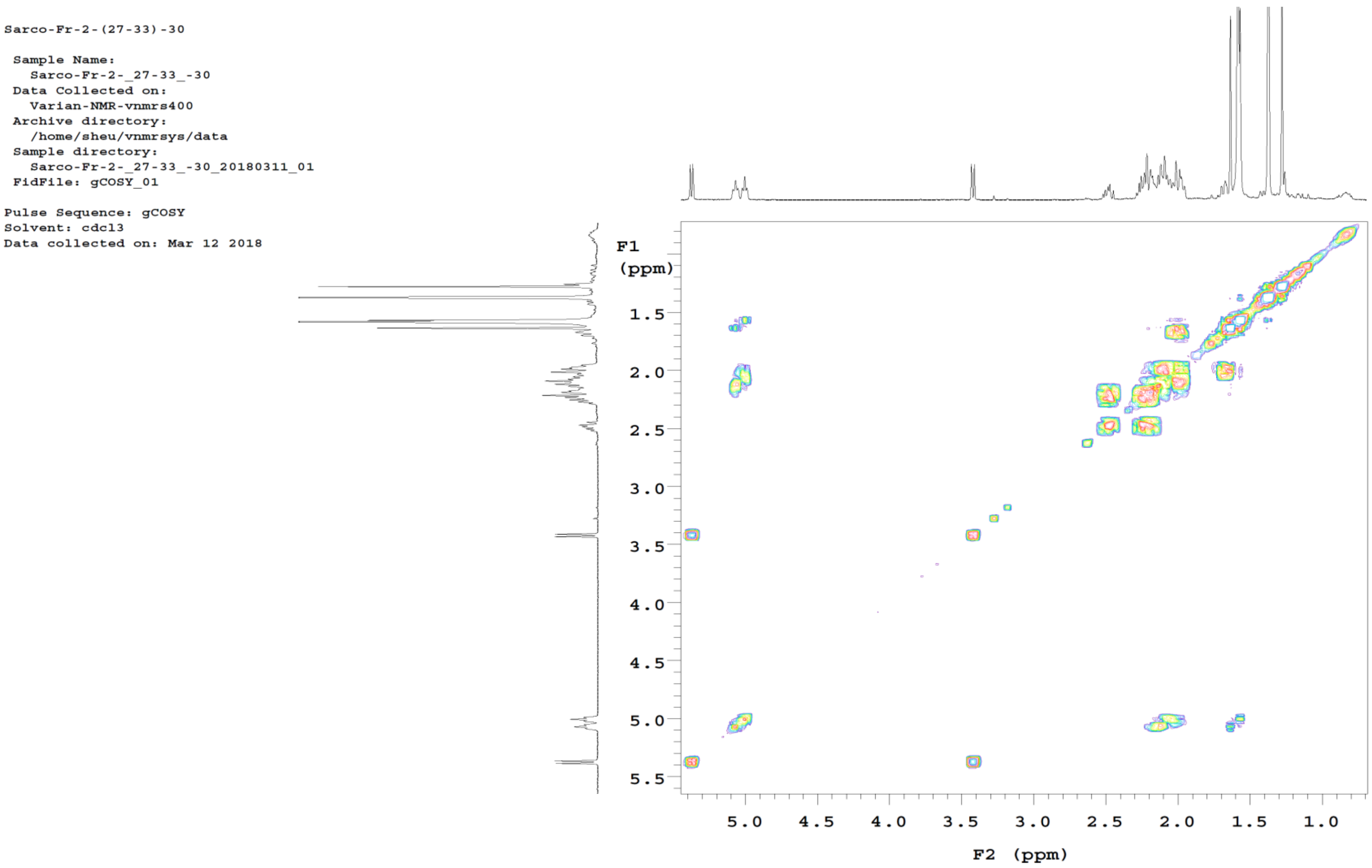

Figure S20: COSY spectrum of **2**

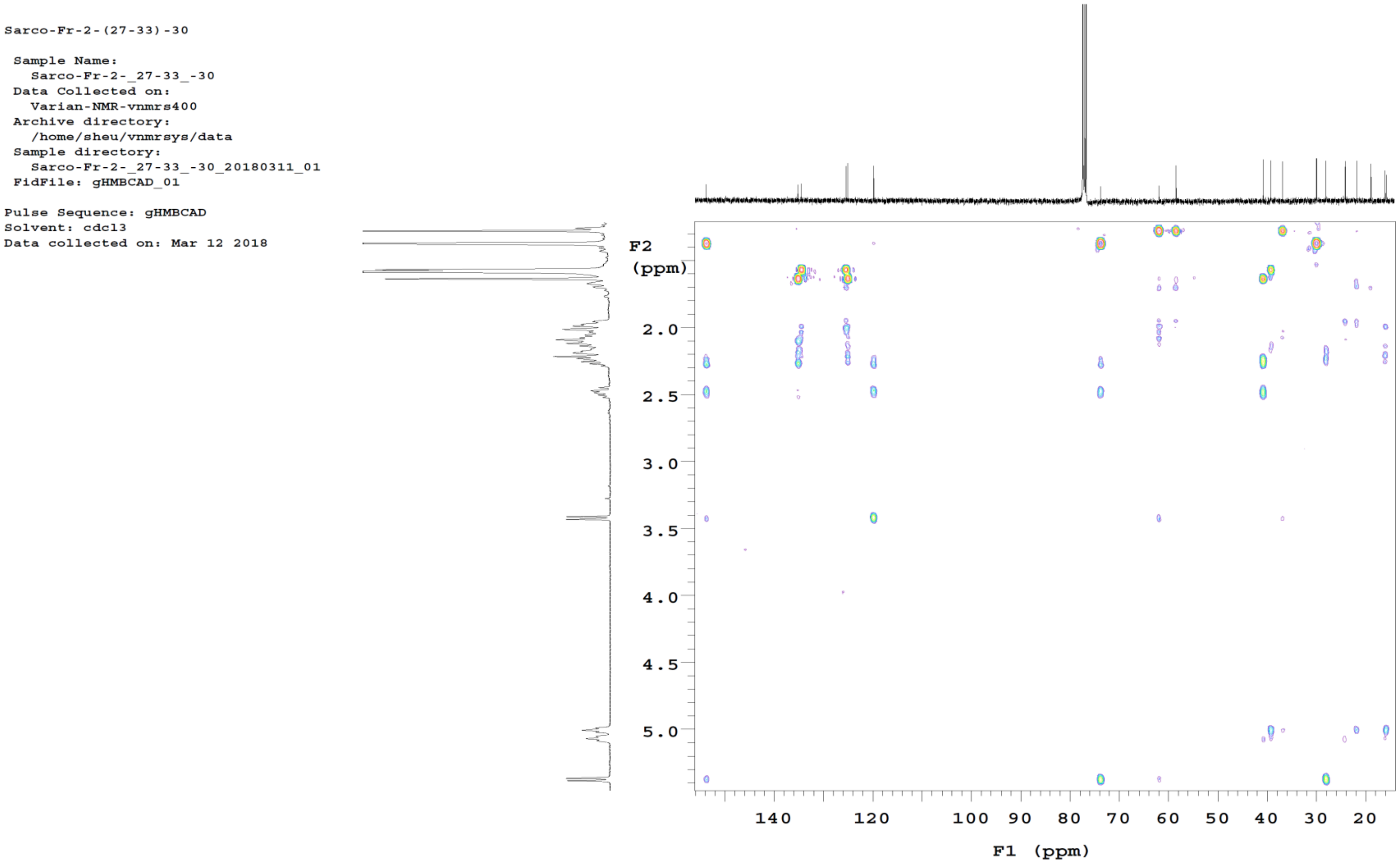

Figure S21: HMBC spectrum of **2**

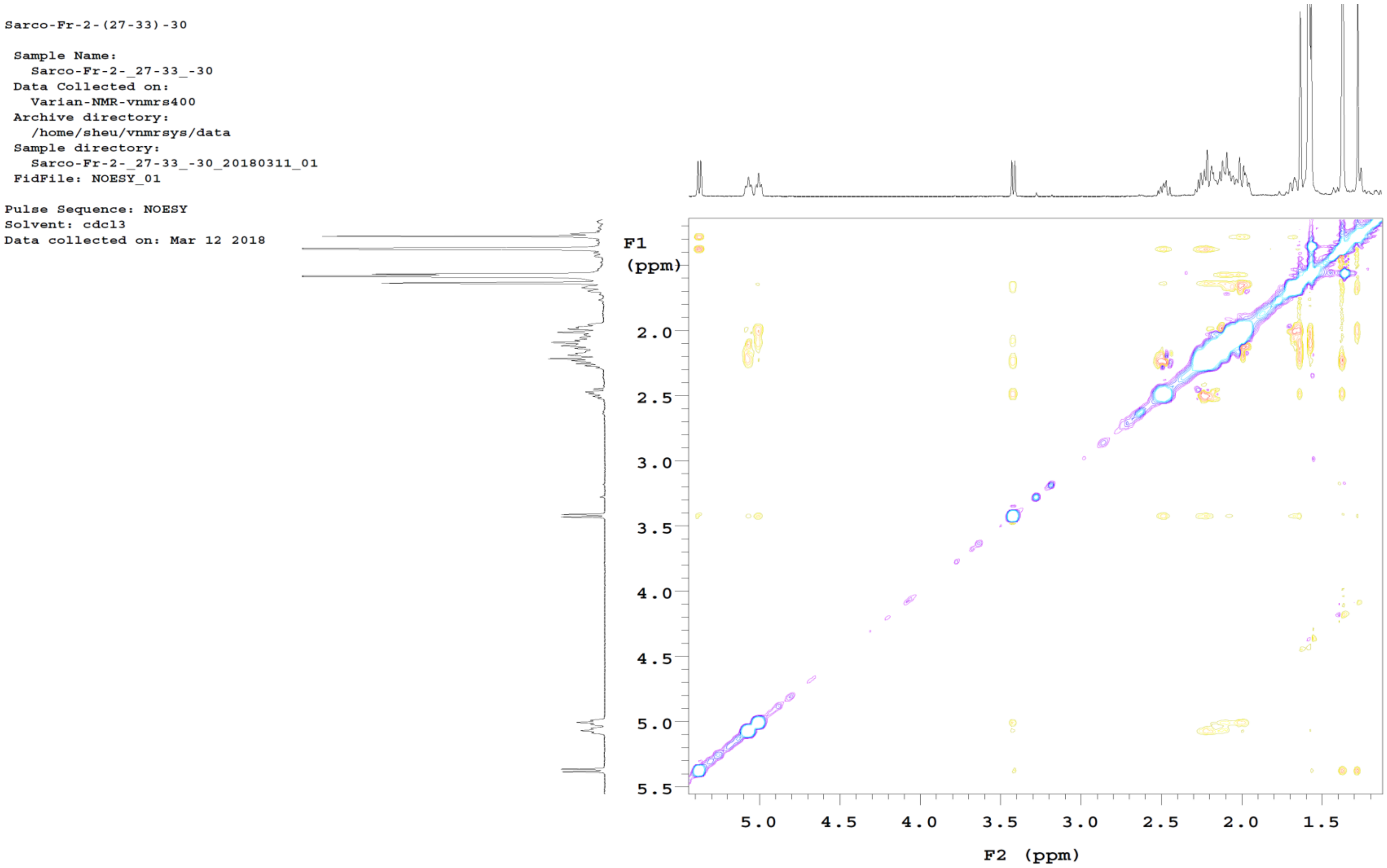

Figure S22: NOESY spectrum of **2**

Compound **3** spectroscopic data

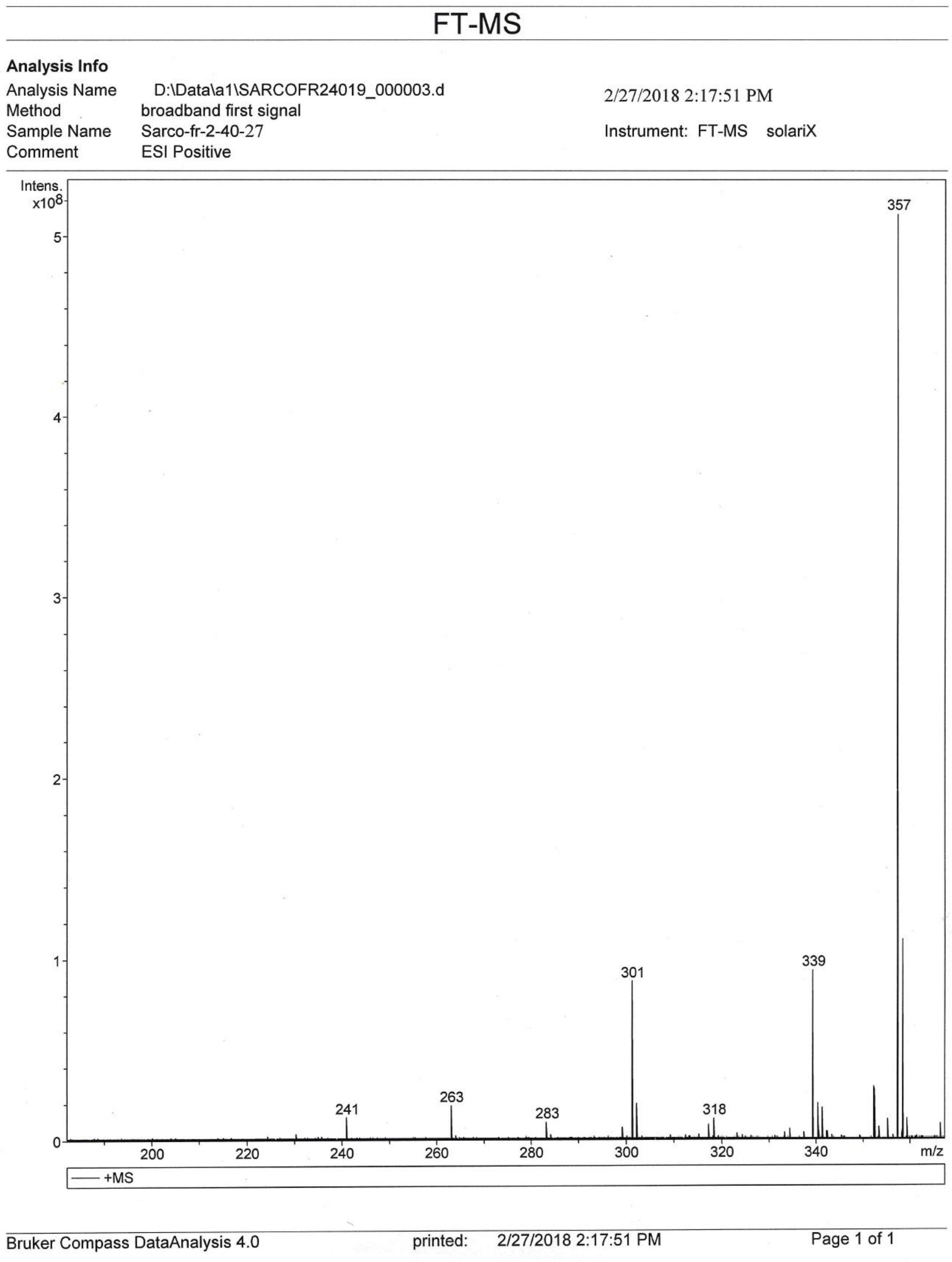

Figure 23: ESIMS spectrum of **3**

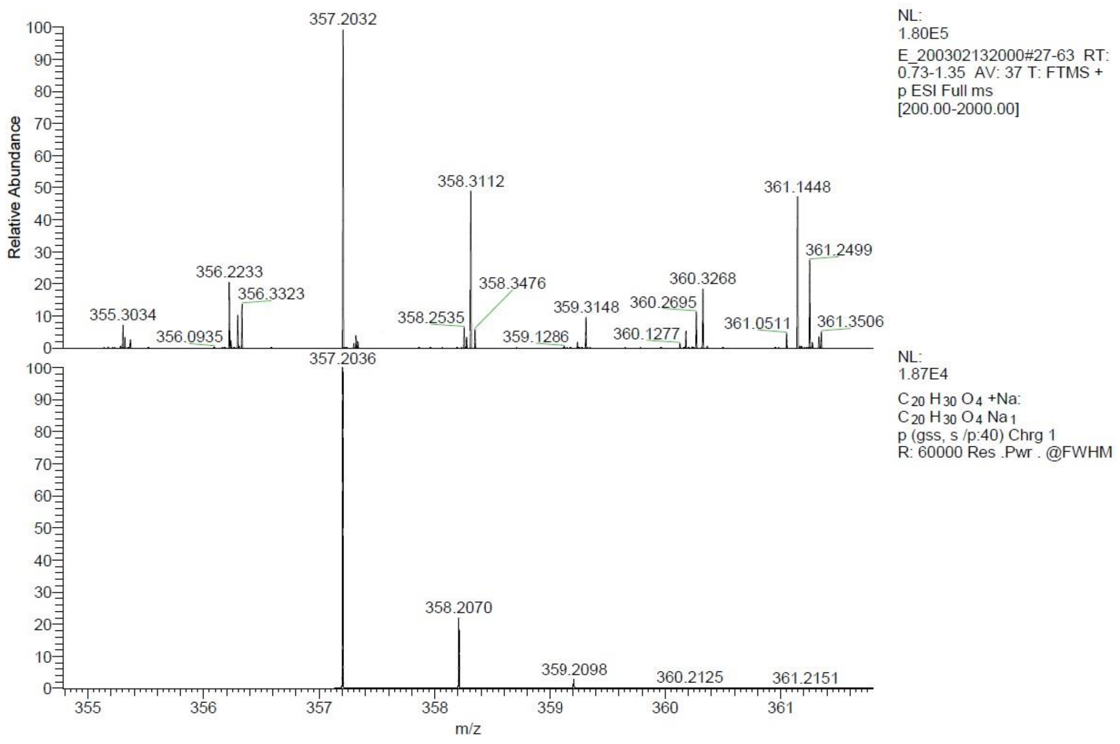

Figure S24: HRESIMS spectrum of **3**

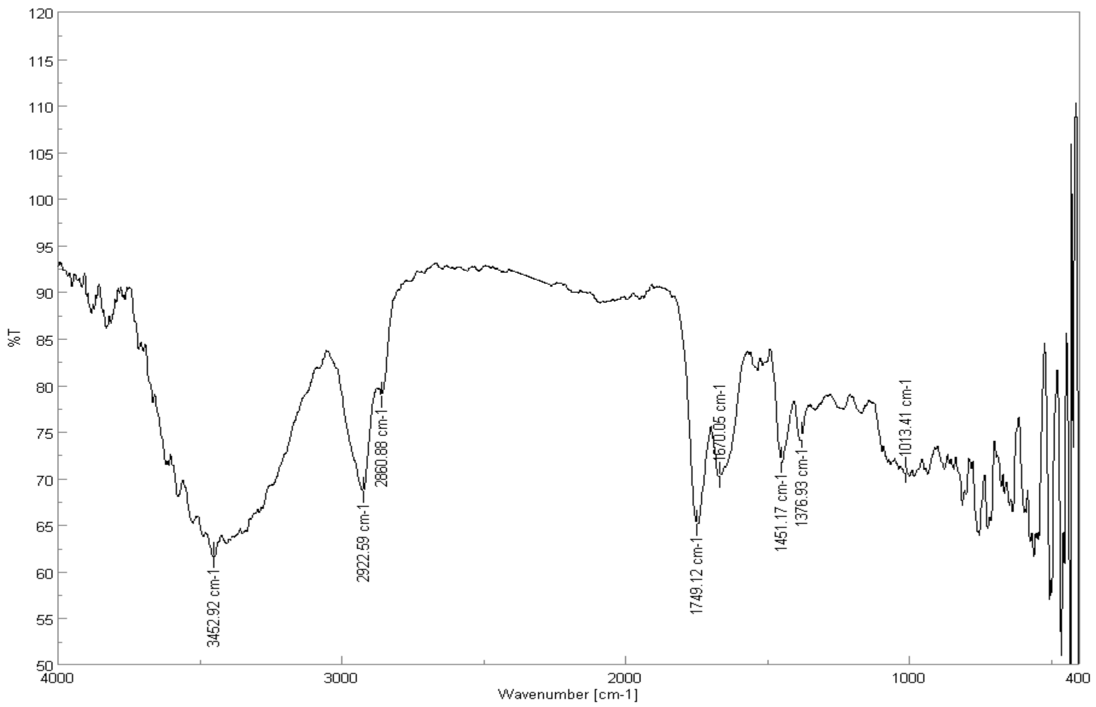

Figure S25: IR spectrum of **3**

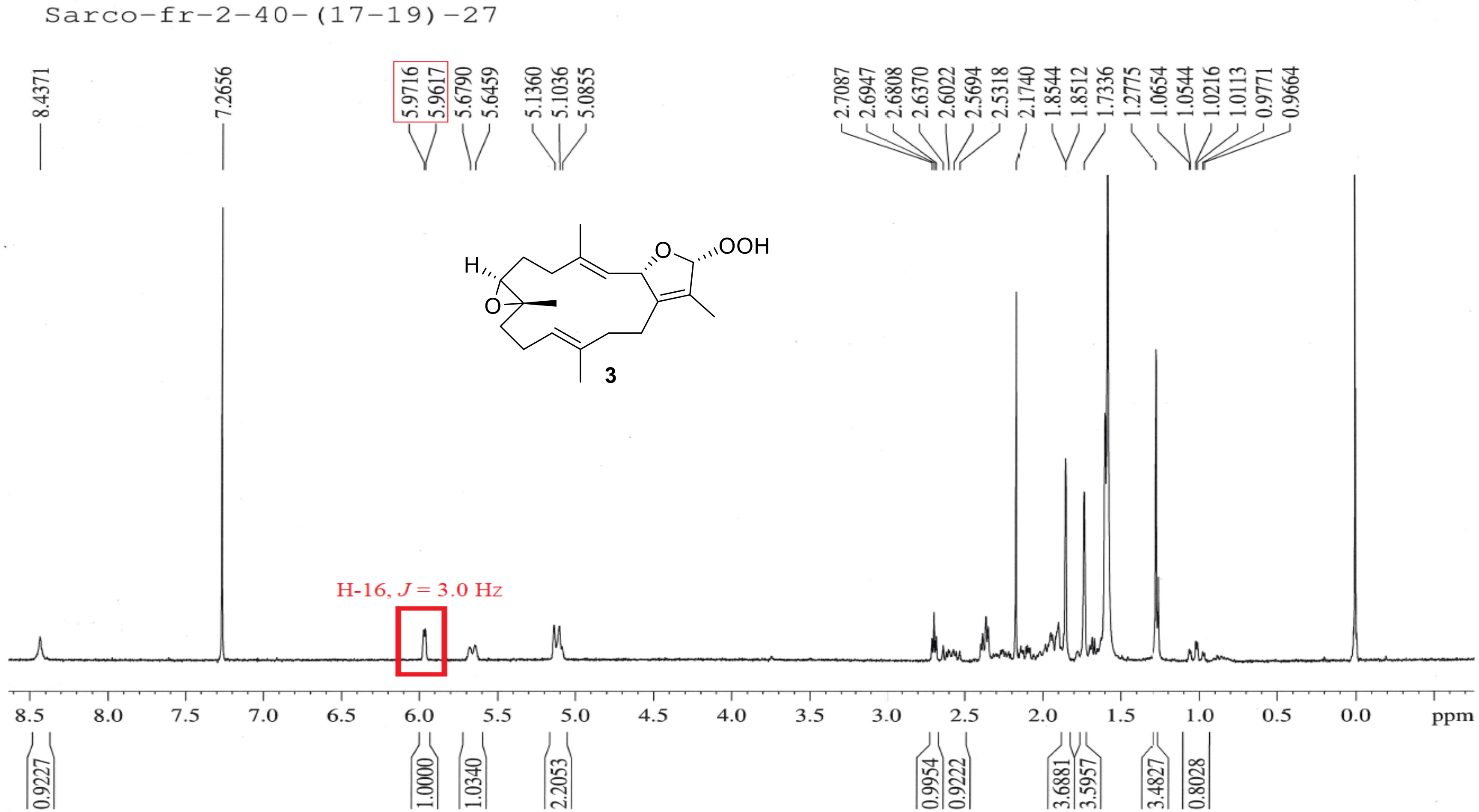

Figure S26: ^{1}H NMR spectrum of **3** in $CDCl_3$ at 300 MHz

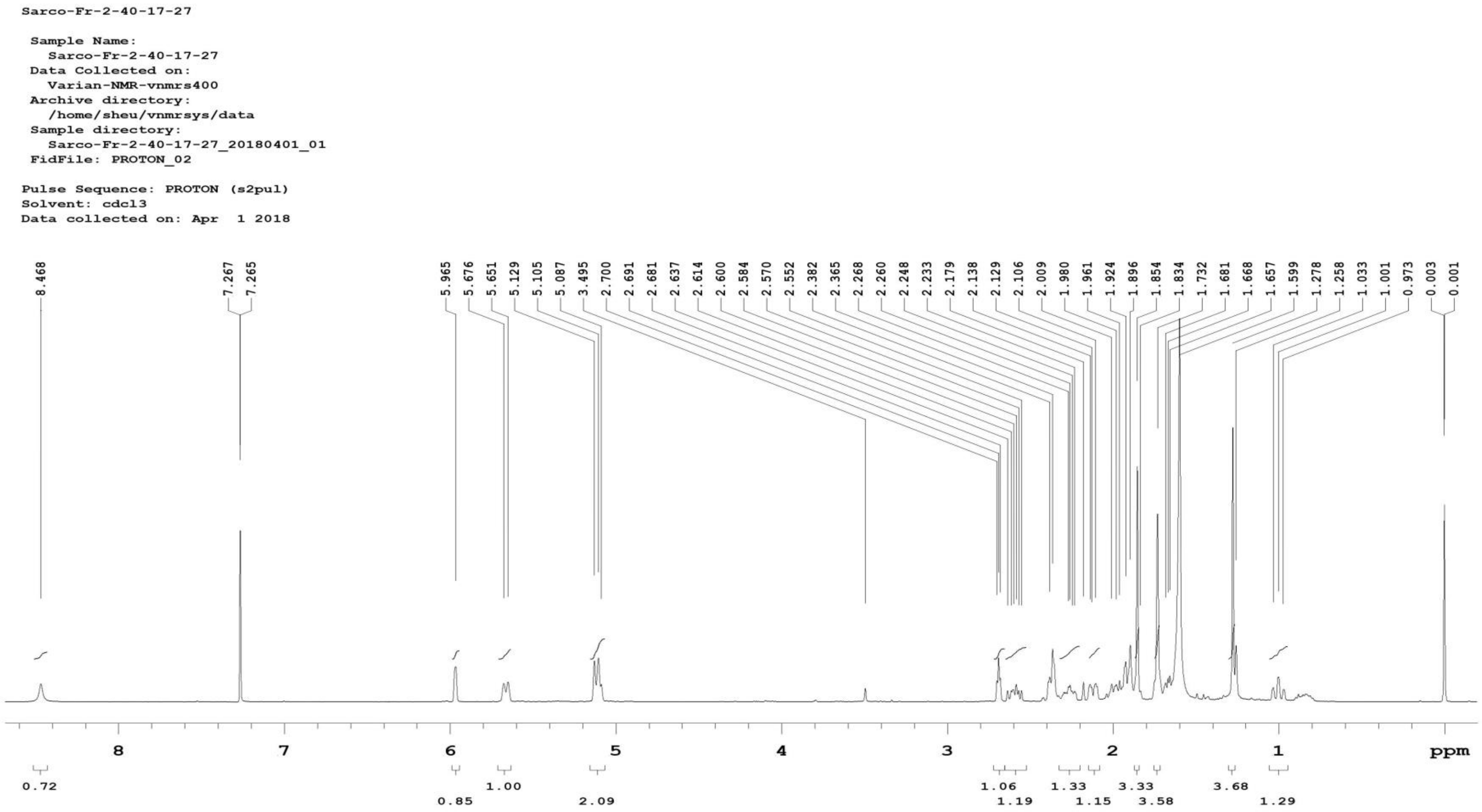

Figure S27: ^{1}H NMR spectrum of **3** in $CDCl_3$ at 400 MHz

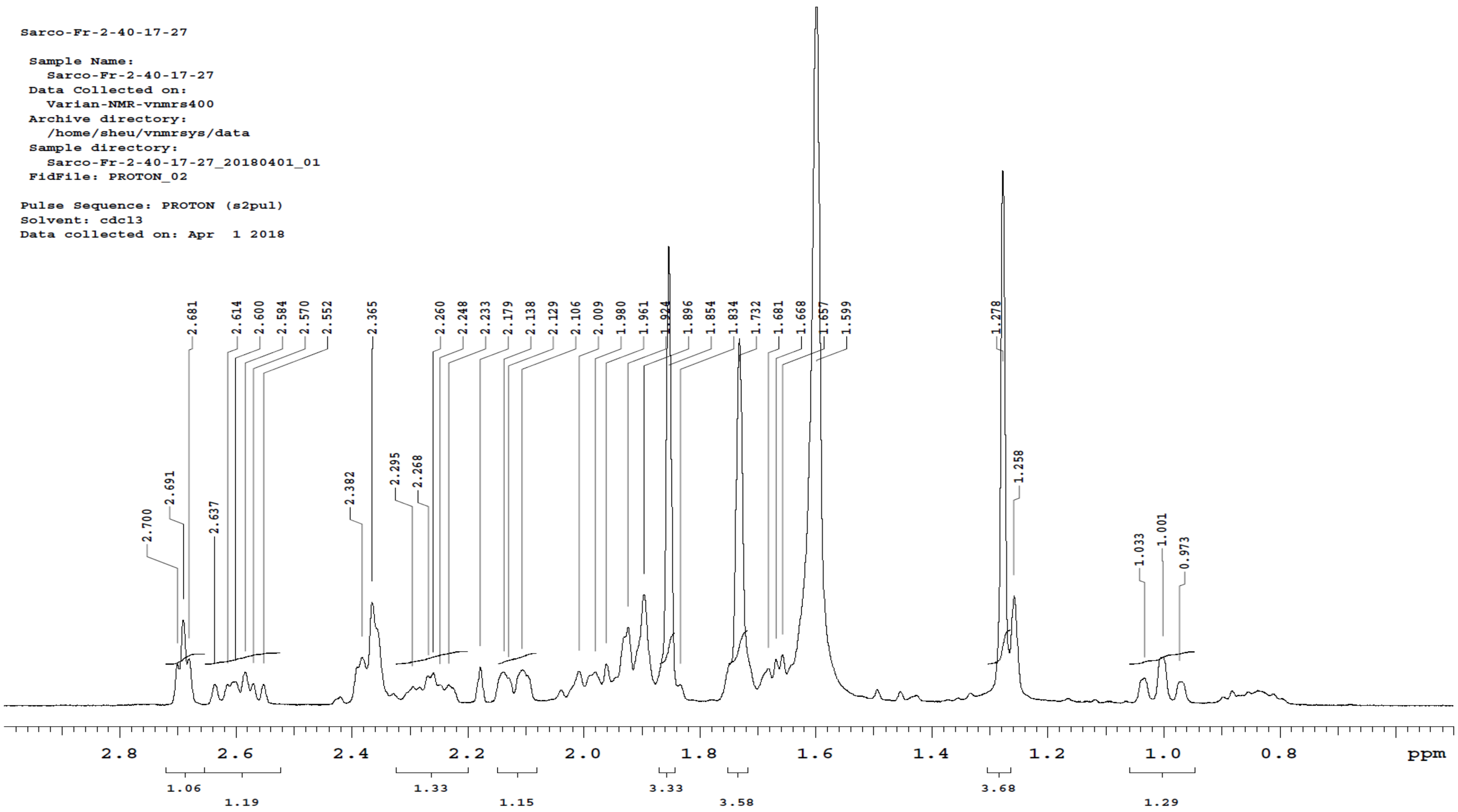

Figure S28: ^{1}H NMR spectrum (from 0.5 to 3.0 ppm) of **3** in $CDCl_3$ at 400 MHz

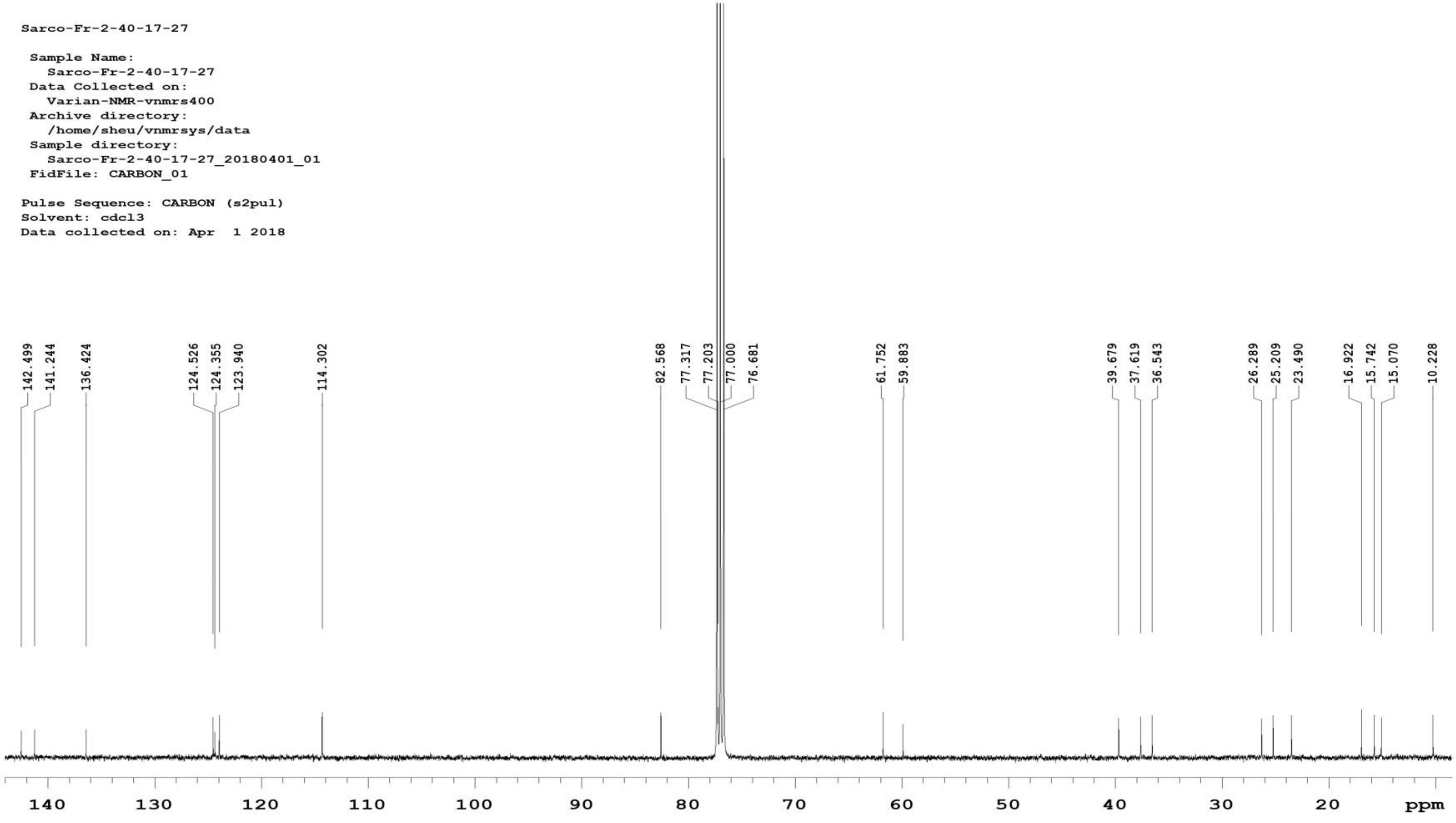

Figure S29: ^{13}C NMR spectrum of **3** in $CDCl_3$ at 100 MHz

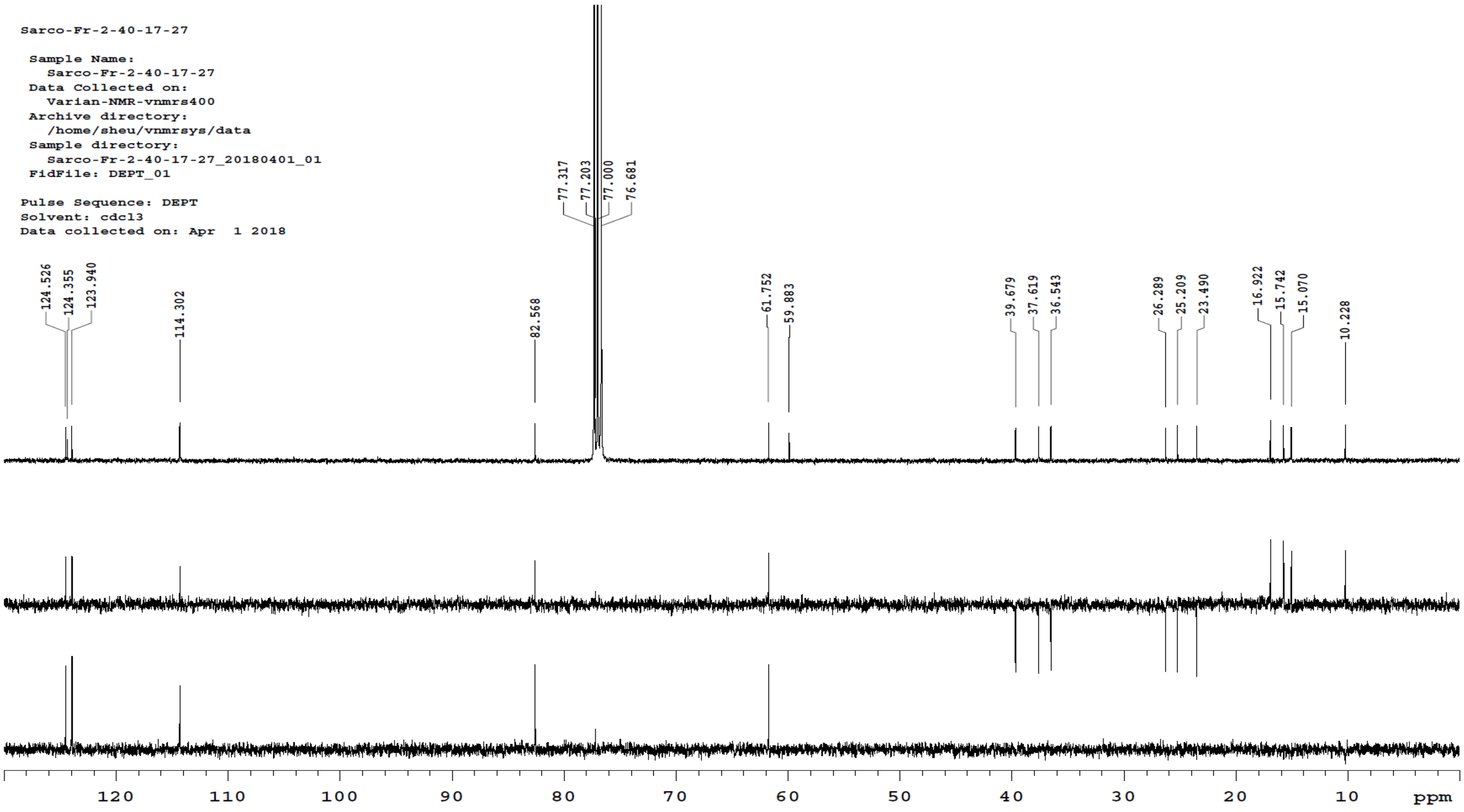

Figure S30: DEPT spectrum of **3**

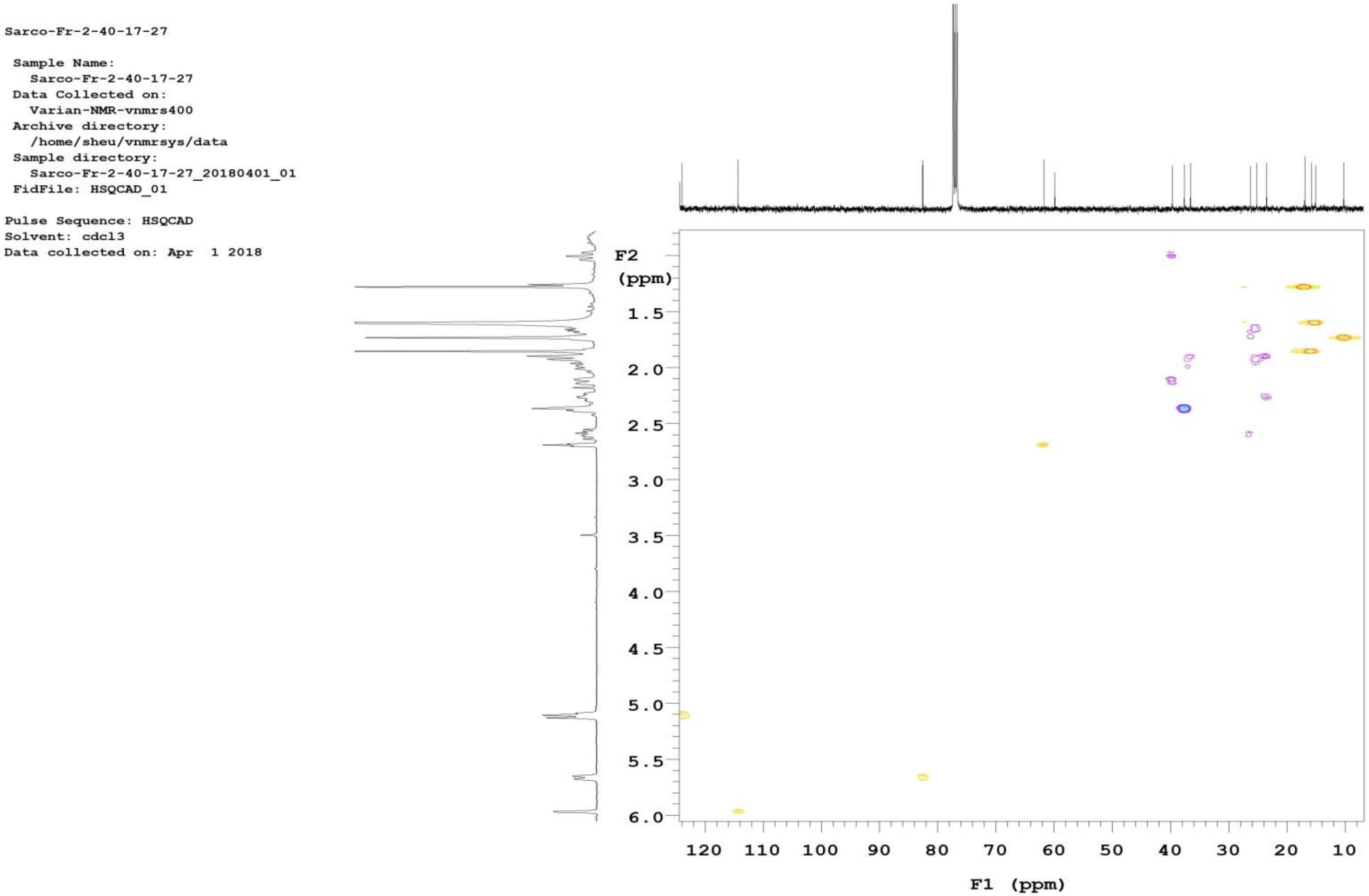

Figure S31: HSQC spectrum of **3**

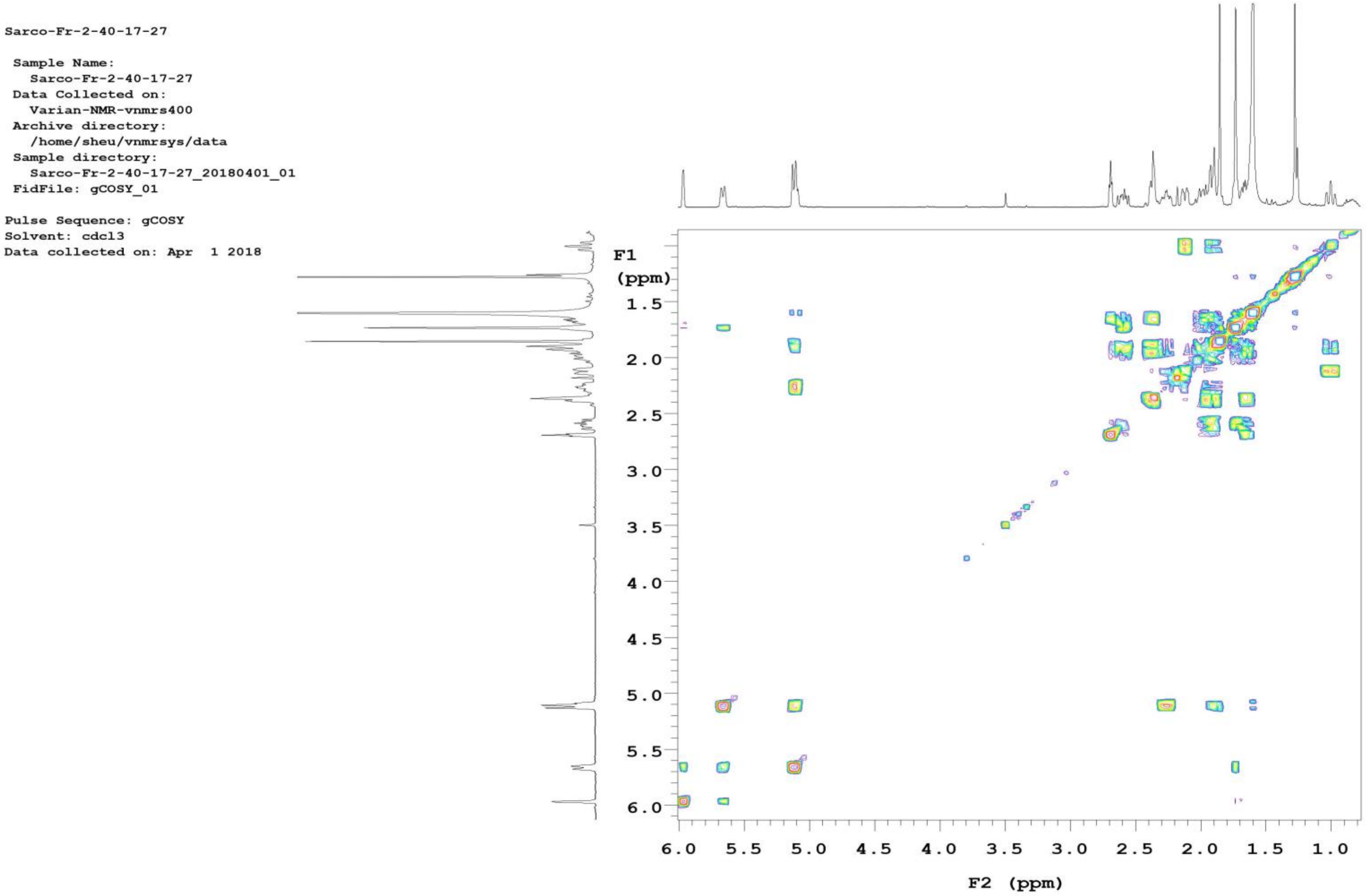

Figure S32: COSY spectrum of **3**

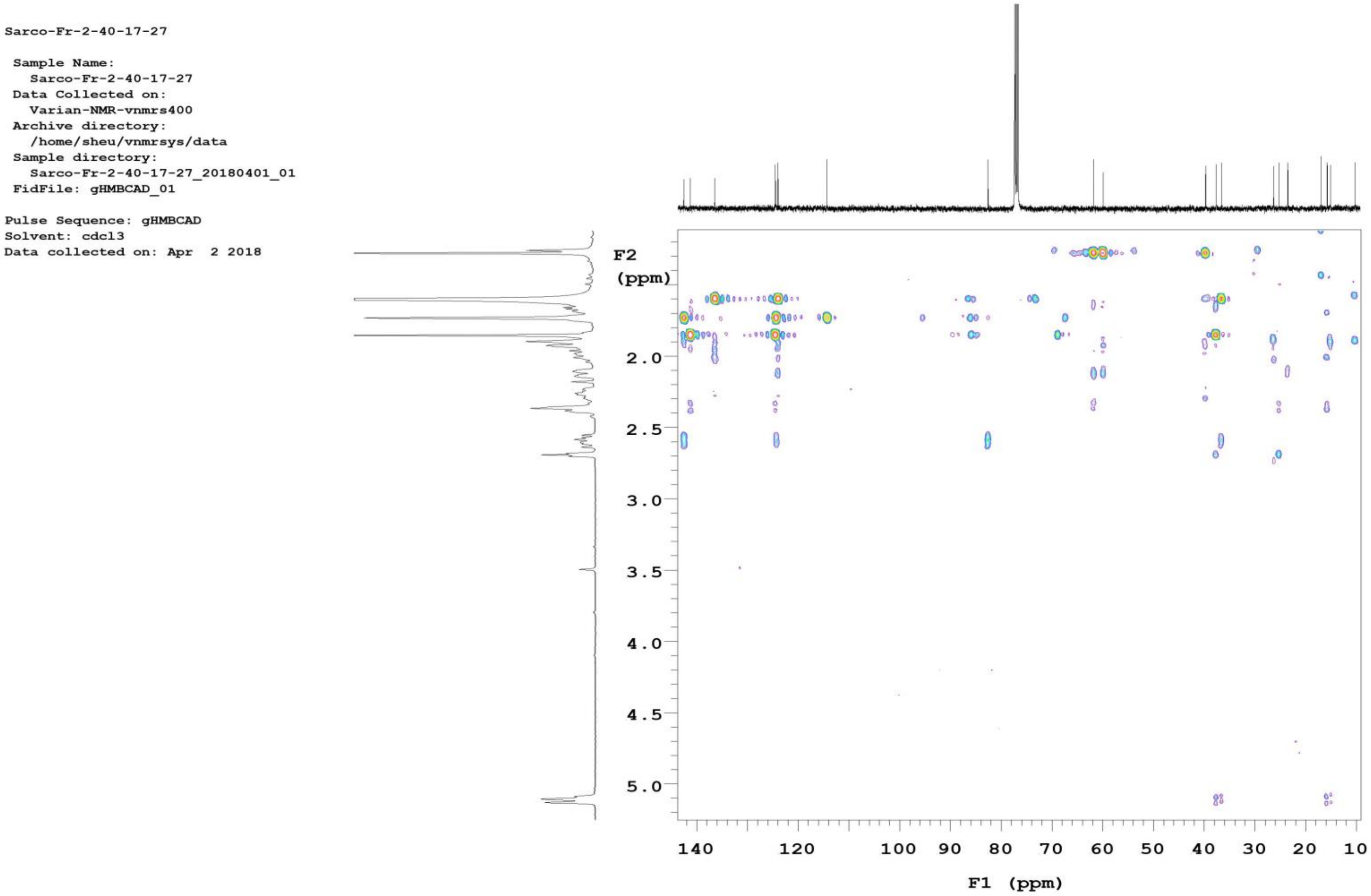

Figure S33: HMBC spectrum of **3**

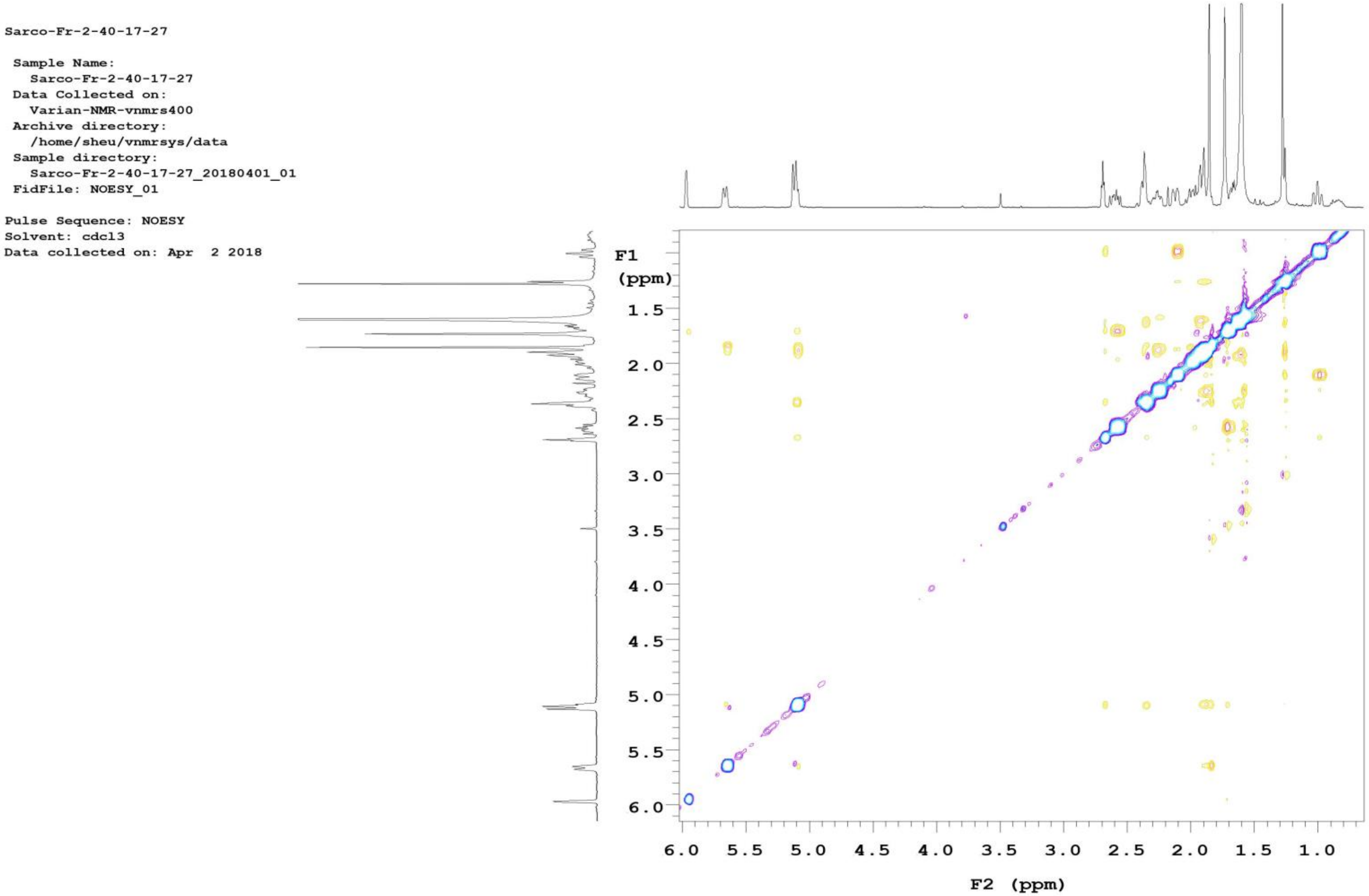

Figure S34: NOESY spectrum of **3**

Compound **4** spectroscopic data

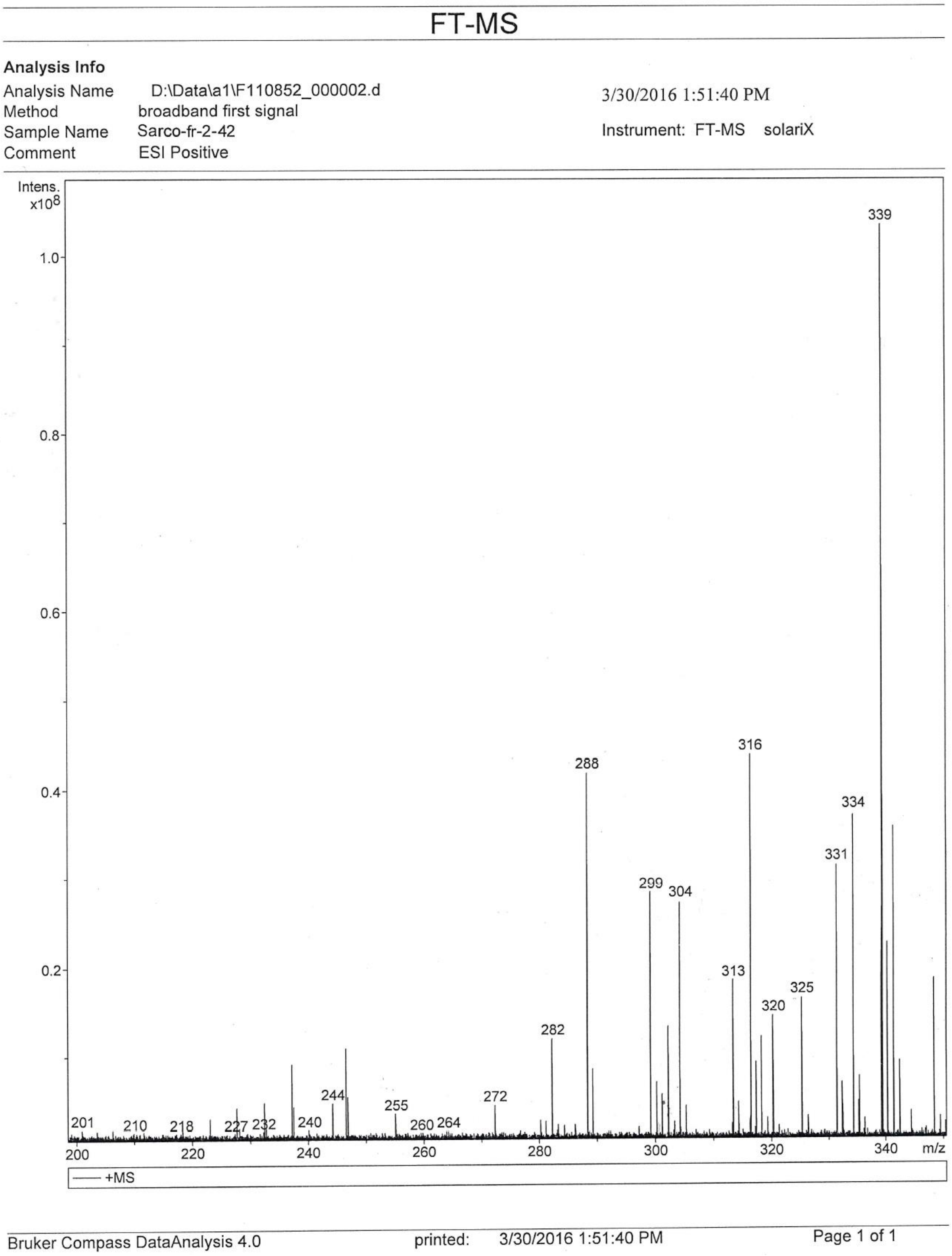

Figure S35: ESIMS spectrum of **4**

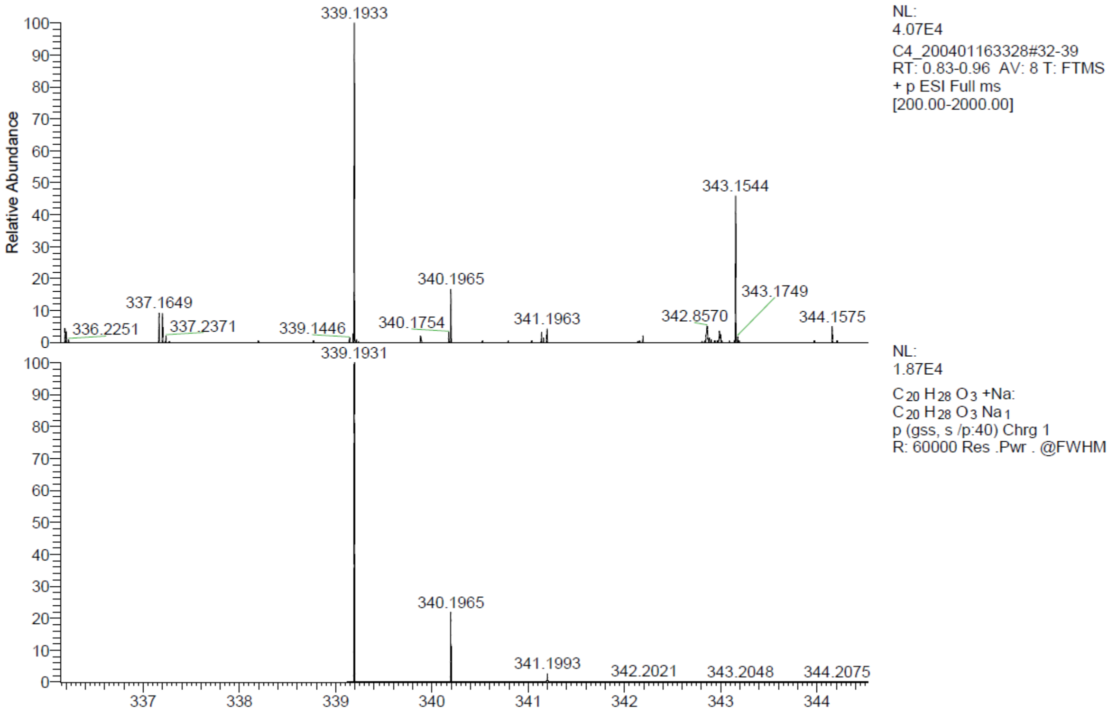

Figure S36: HRESIMS spectrum of **4**

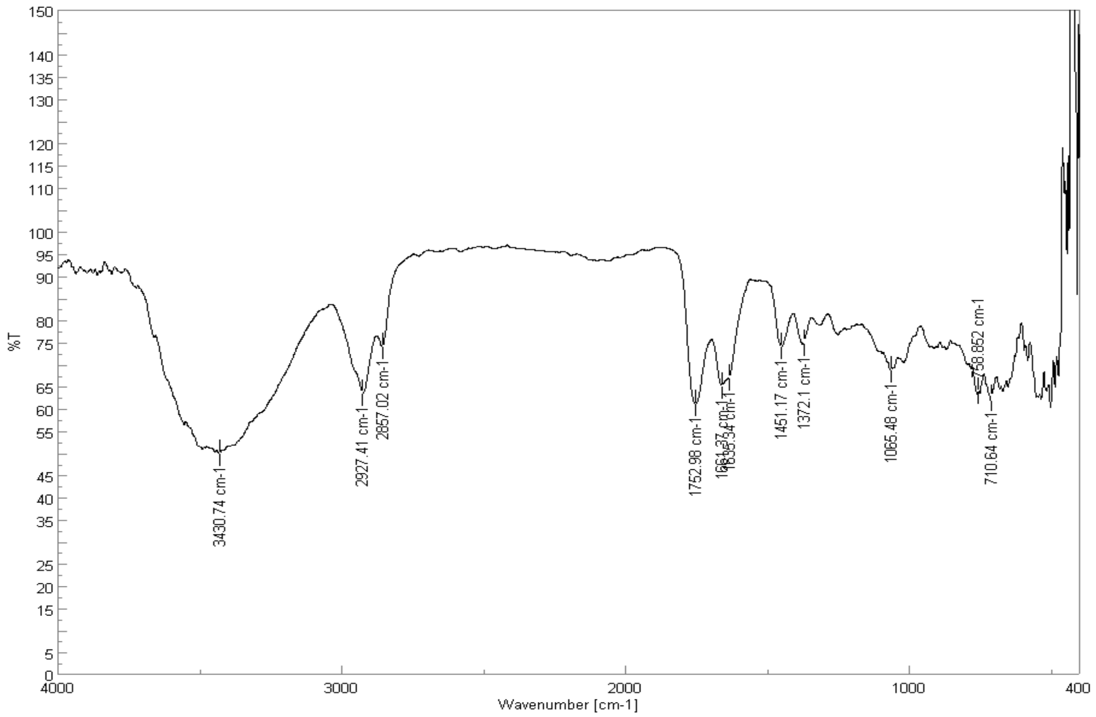

Figure S37: IR spectrum of **4**

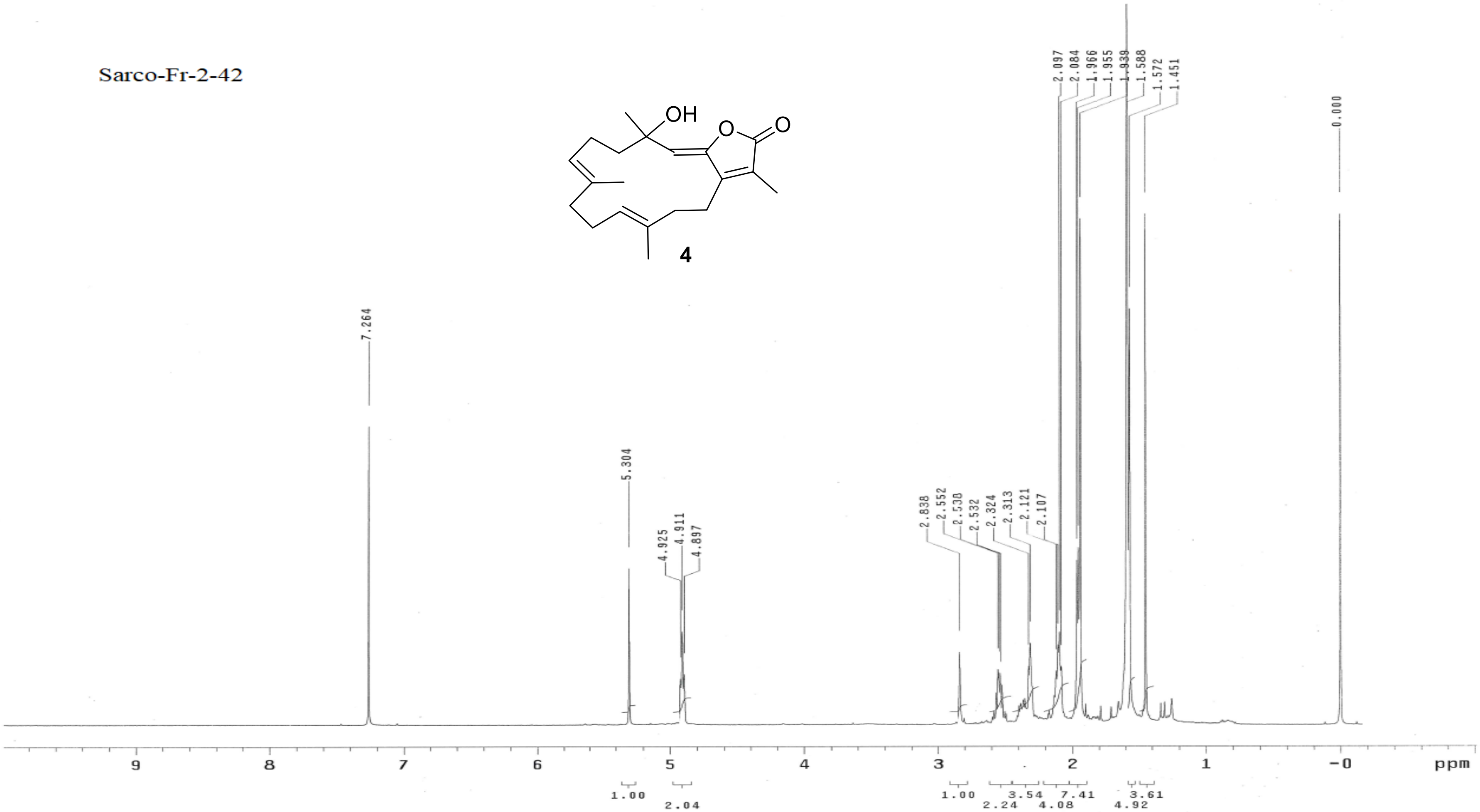

Figure S38: ^{1}H NMR spectrum of **4** in $CDCl_3$ at 500 MHz

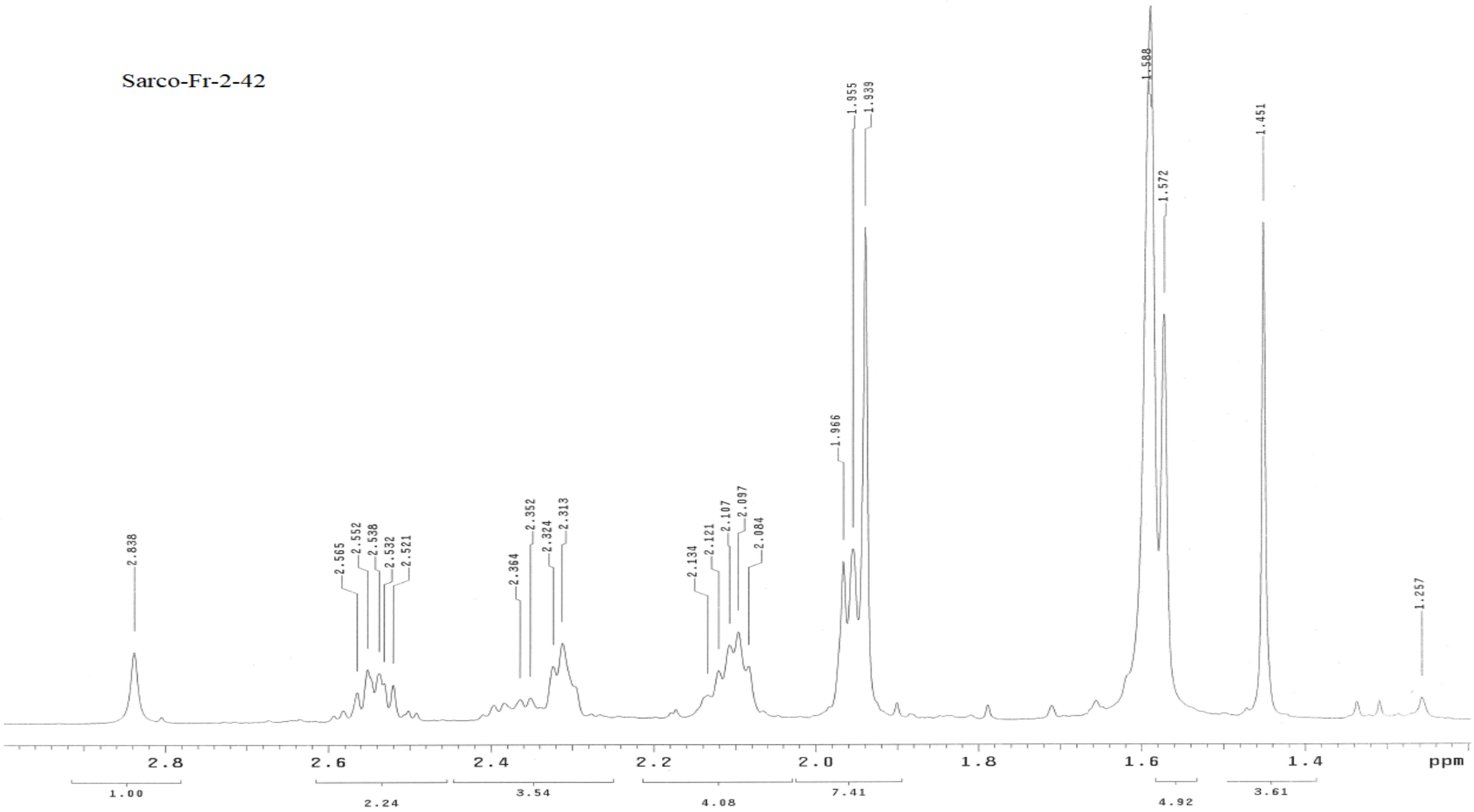

Figure S39: ^{1}H NMR spectrum (from 1.2 to 3.0 ppm) of **4** in $CDCl_3$ at 500 MHz

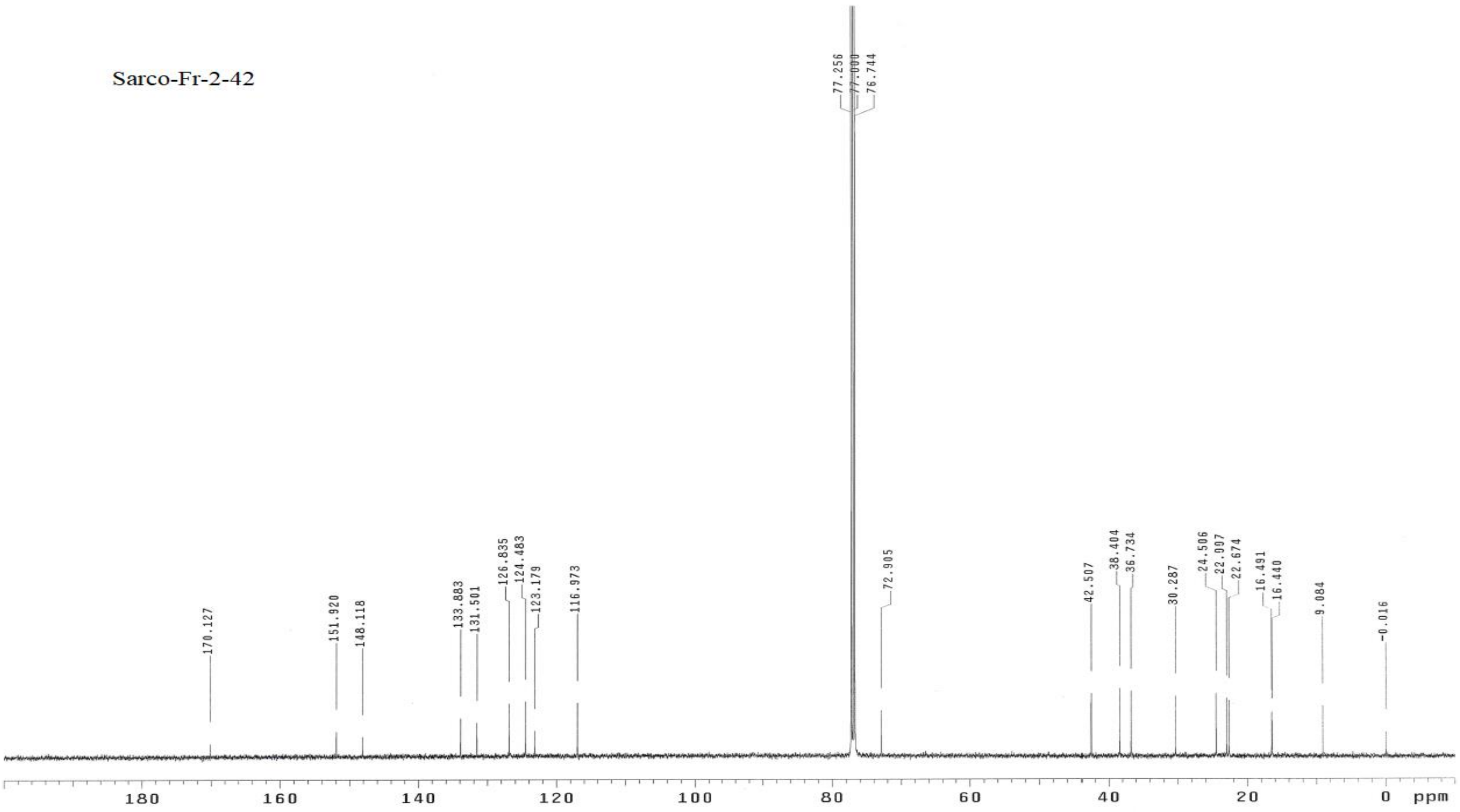

Figure S40: ^{13}C NMR spectrum of **4** in $CDCl_3$ at 125 MHz

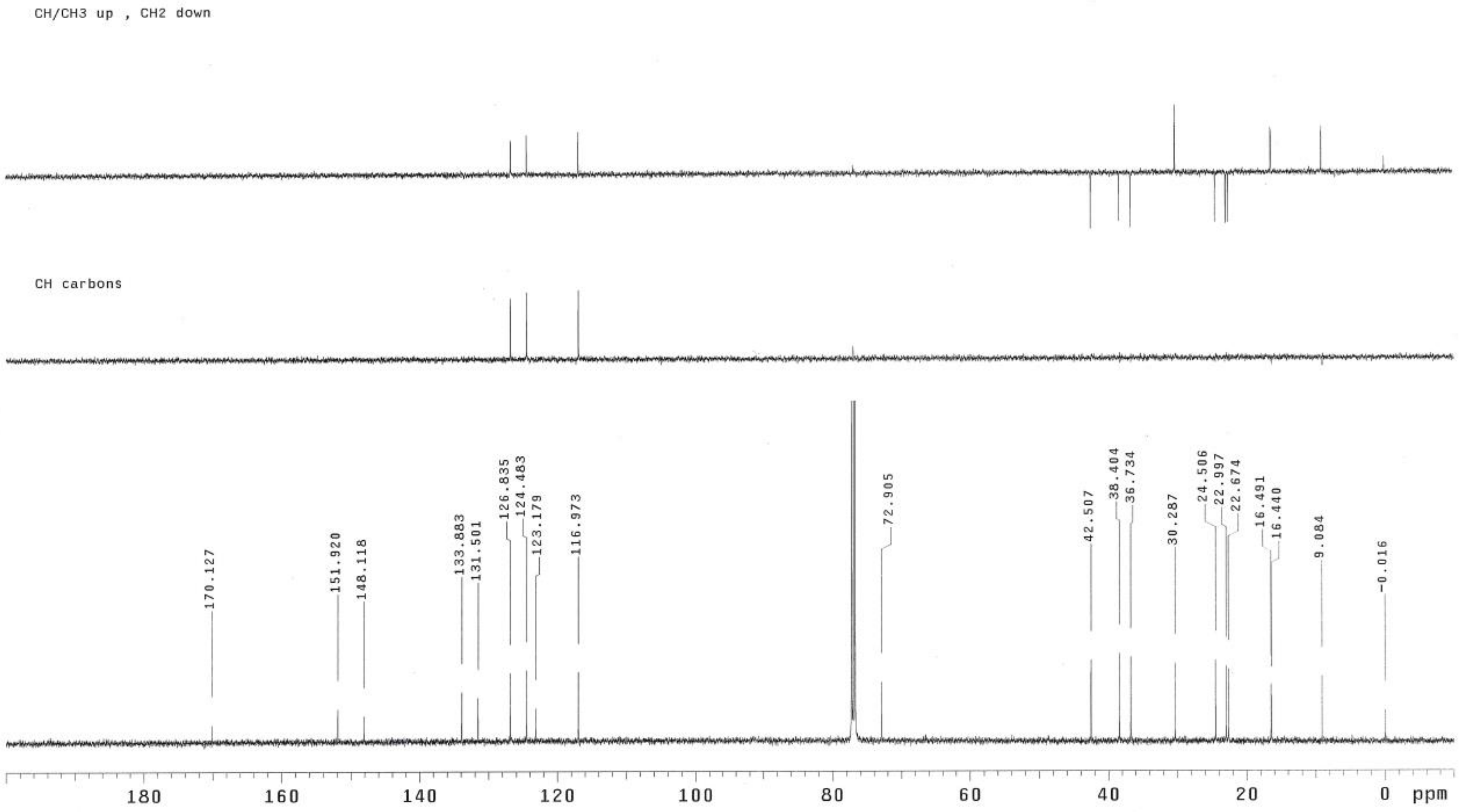

Figure S41: DEPT spectrum of **4**

Sarco-Fr-2-42

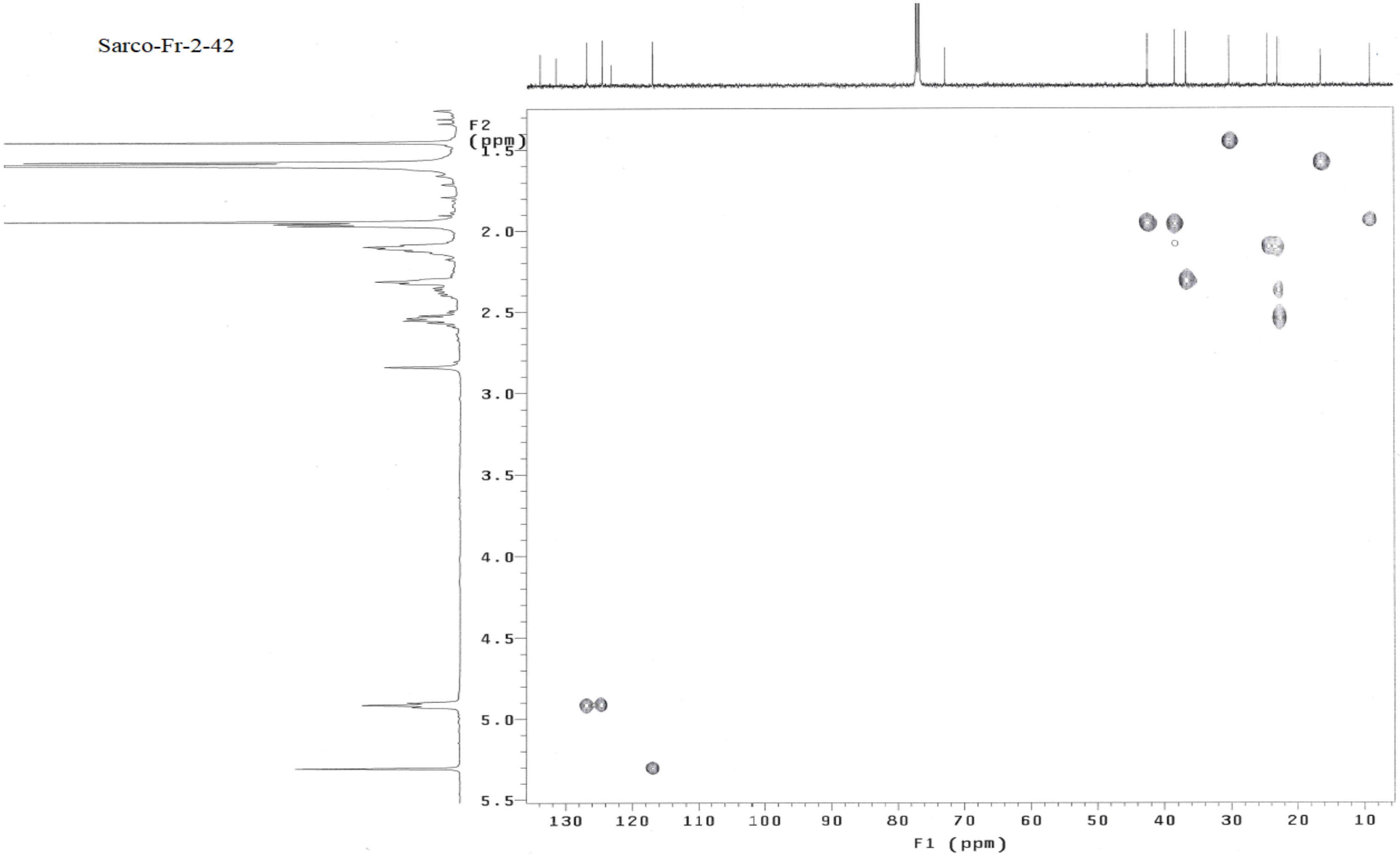

Figure S42: HSQC spectrum of **4**

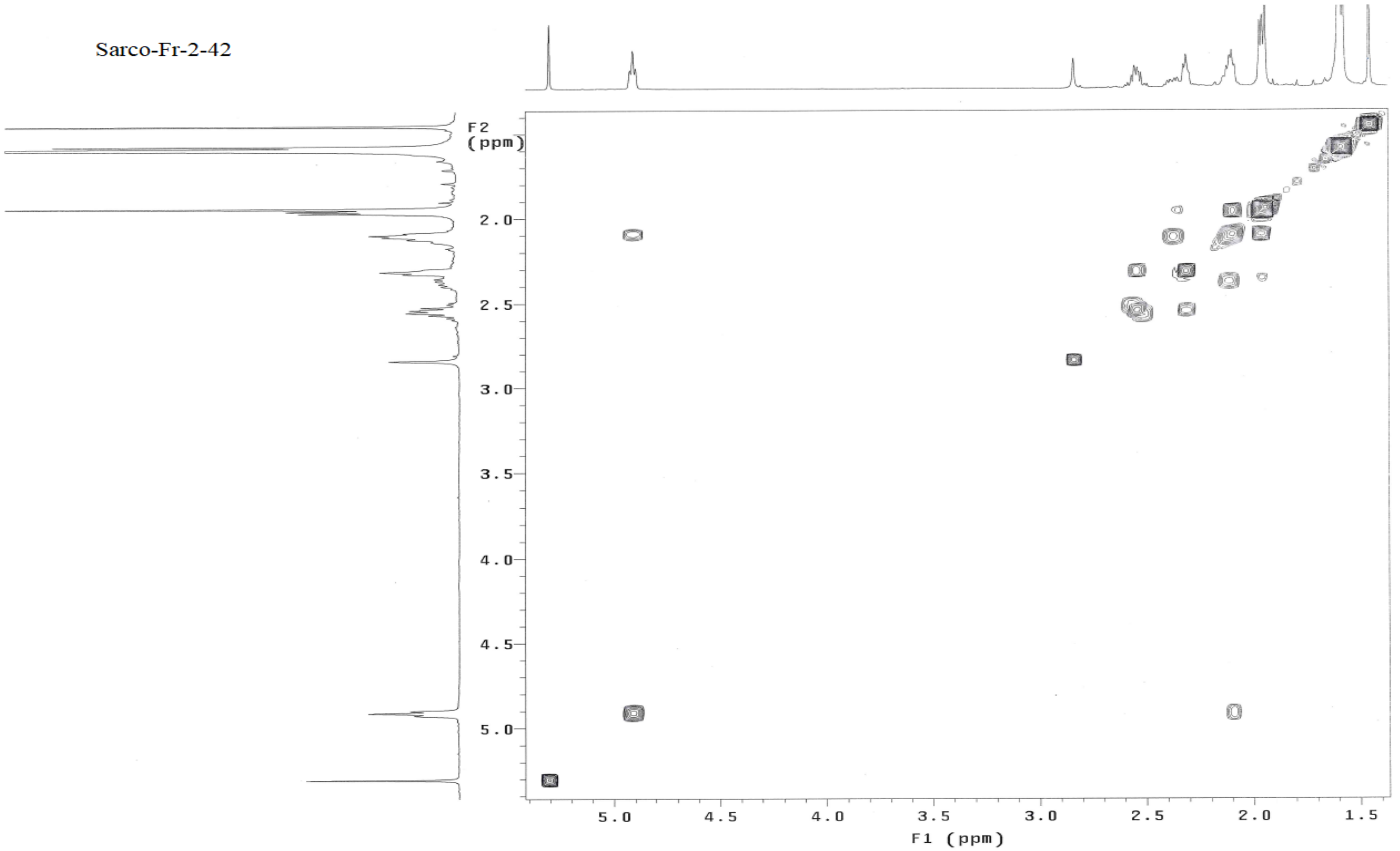

Figure S43: COSY spectrum of **4**

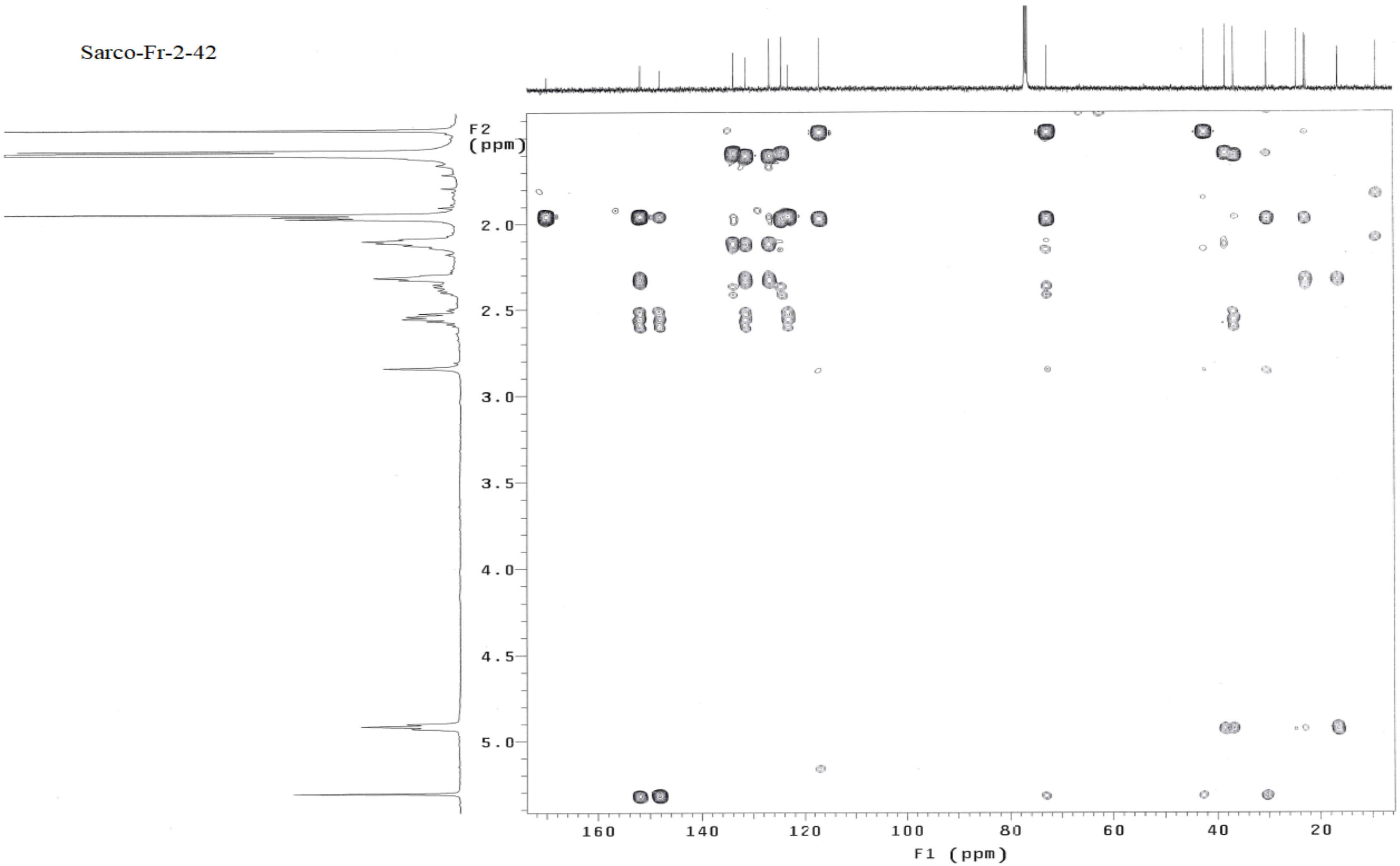

Figure S44: HMBC spectrum of **4**

Sarco-Fr-2-42

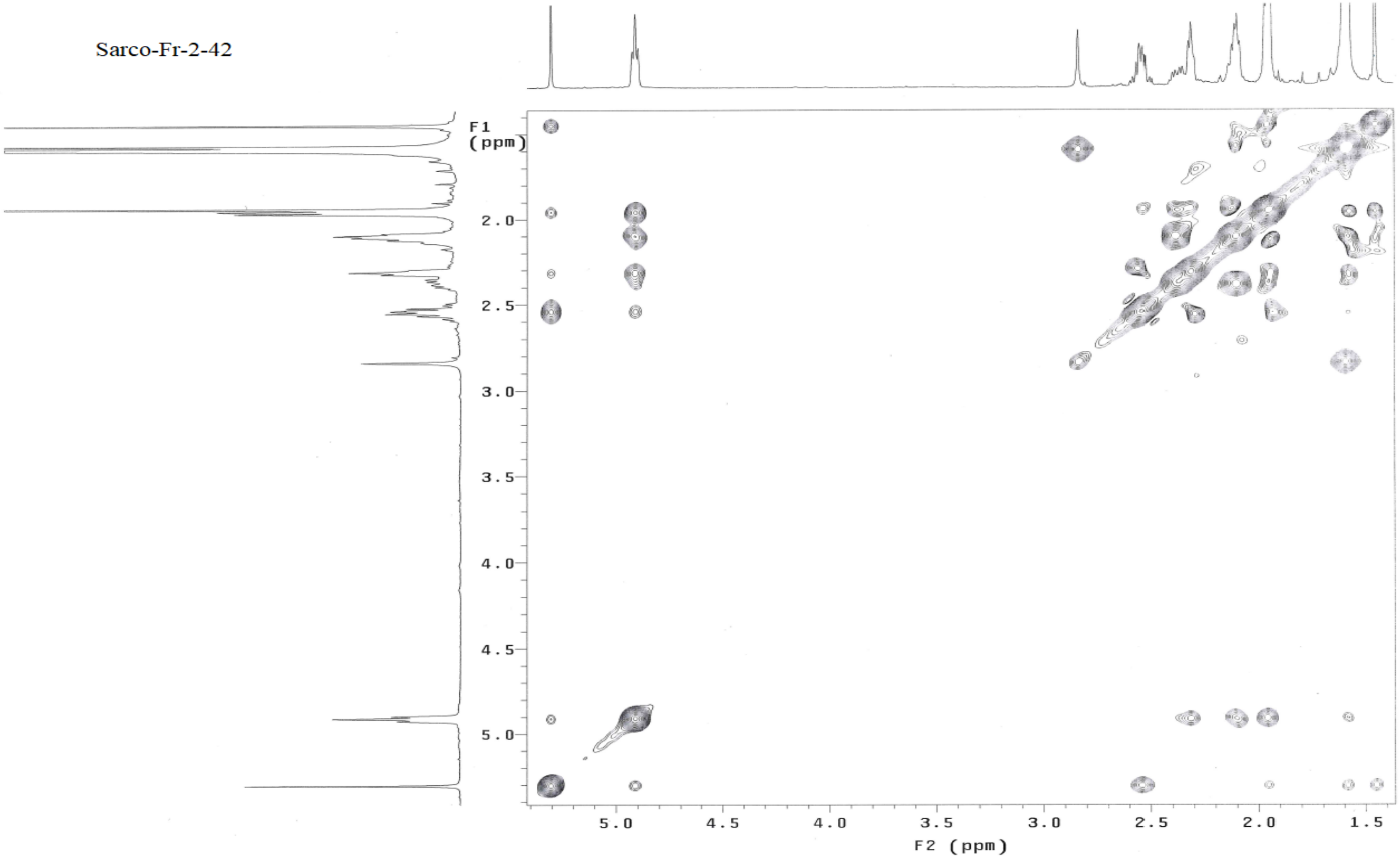

Figure S45: NOESY spectrum of **4**
